# Microbiota, not host origin drives *ex vivo* intestinal epithelial responses

**DOI:** 10.1080/19490976.2022.2089003

**Published:** 2022-06-26

**Authors:** Kaline Arnauts, Padhmanand Sudhakar, Sare Verstockt, Cynthia Lapierre, Selina Potche, Clara Caenepeel, Bram Verstockt, Jeroen Raes, Séverine Vermeire, João Sabino, Catherine Verfaillie, Marc Ferrante

**Affiliations:** a Department of Chronic Diseases, Metabolism and Ageing (CHROMETA), Translational Research Center for Gastrointestinal Disorders (TARGID), KU Leuven, Leuven, Belgium; b Department of Development and Regeneration, Stem Cell Institute Leuven (SCIL), KU Leuven, Leuven, Belgium; c Department of Gastroenterology and Hepatology, University Hospitals Leuven, KU Leuven, Leuven, Belgium; d Department of Microbiology and Immunology, Rega Institute, KU Leuven, Leuven, Belgium

**Keywords:** Ulcerative colitis, epithelial cells, microbiota, dysbiosis, organoids

## Abstract

Microbial dysbiosis is an established finding in patients with inflammatory bowel disease (IBD), but host-microbial interactions are poorly understood. We aimed to unravel the effect of microbiota exposure on intestinal epithelial cells. Confluent Transwell® organoid monolayers of eight UC patients and eight non-IBD controls were co-cultured for six hours with microbiota (3x10^8^ cells) of UC patients or a healthy volunteer (HV), in the presence or absence of an inflammatory cytokine mix. Transepithelial electrical resistance (TEER), fluorescein isothiocyanate (FITC) dextran measurements, and RNA sequencing were performed on epithelial cells, and 16S rRNA sequencing on microbiota samples before and after co-culture. Transcriptomic response following microbiota exposure was not different between epithelial cells from UC patients or non-IBD controls. Following UC microbiota exposure, but not HV microbiota, a strong decrease in epithelial barrier integrity was observed in both UC and HV epithelial cells by TEER and FITC dextran measurements. Exposure of inflamed epithelium to UC microbiota induced transcriptomic stress pathways including activation of EGR1, MAPK and JAK/STAT signaling, as well as AP-1 family and FOSL transcripts. Stress responses after HV microbiota stimulation were milder. We conclude that not the epithelial cell origin (UC versus non-IBD) but the microbial donor drives transcriptomic responses, as exposure to UC microbiota was sufficient to induce stress responses in all epithelial cells. Further research on therapies to restore the microbial balance, to remove the constant trigger of dysbiosis, is required.

## Graphical abstract

**Figure:**
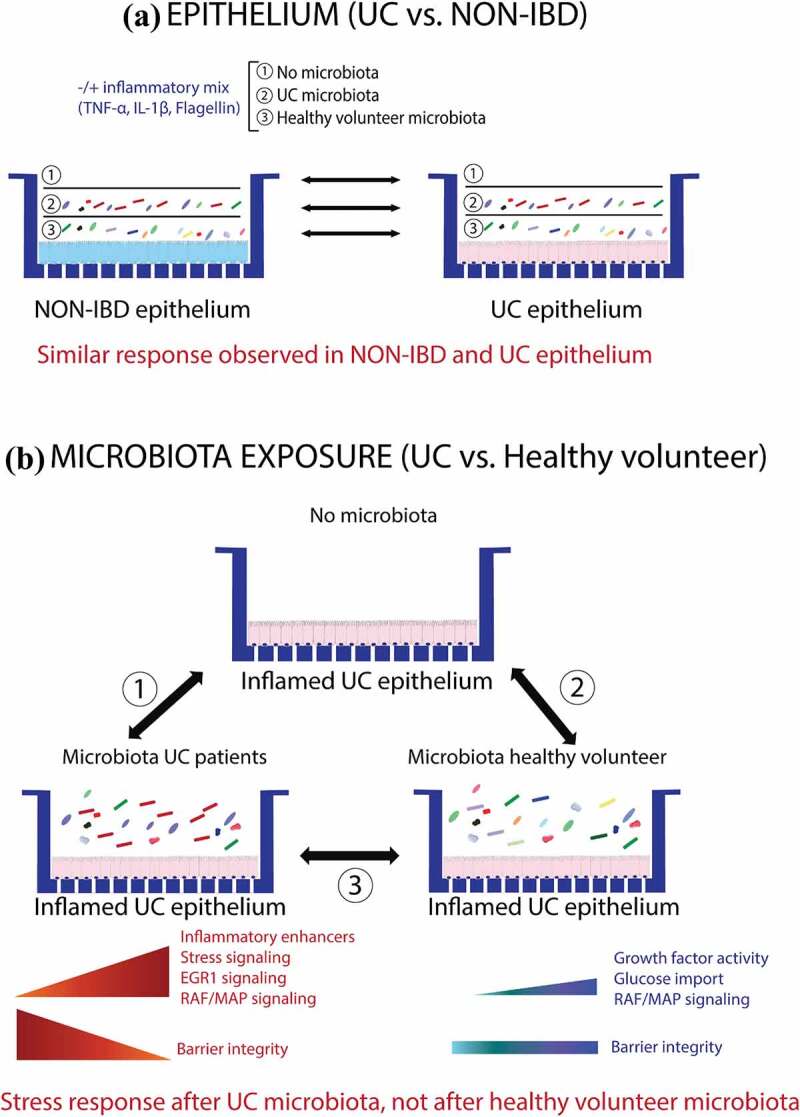

## 1. Introduction

Inflammatory bowel disease (IBD) is a chronic inflammatory disorder of the gastrointestinal (GI) tract of unknown etiology and comprises two main entities, Crohn’s disease (CD) and ulcerative colitis (UC). In addition to a complex interplay between genetic predisposition, environmental factors and a dysregulated immune system, alterations in the gut microbiota have been associated with the onset and exacerbation of IBD.^1^

A symbiotic microbiota is essential for vitamin and iron metabolism, immune maturation, epithelial barrier function, pathogen displacement and metabolic homeostasis. Alterations in the microbiota composition, classically called “dysbiosis”, have been linked to multiple disorders including IBD.^2,3^

The correlation between the gut microbiota and IBD has been highlighted in preclinical models in which inflammation could not develop in germ-free mice.^4^ In contrast, transplantation of microbiota from colitis mice to wild-type mice promoted an inflammatory phenotype.^5^ Human studies furthermore underscore the importance of the microbiome in triggering inflammation as fecal stream diversion following ileocolonic resection prevents early recurrence of Crohn’s disease.^6,7^ Compared to a healthy population, the shift in microbiota of IBD patients is characterized by a decrease in both biodiversity and richness.^8–10^ Although dysbiosis in UC patients has been demonstrated repeatedly, effects on intestinal epithelial cells are not understood.

Interactions between microbiota and host take place at the intestinal epithelium. In homeostatic conditions, the intestinal epithelial layer forms a tight physical and chemical protective barrier, preventing contact of harmful bacteria and toxic agents present in the digestive lumen with the underlying tissues. A thick mucus layer is secreted by Goblet cells, while Paneth cells release antimicrobial defensins into this mucus.^11^ In UC, the mucosal barrier is disrupted, resulting in undesired contact of bacteria with intestinal epithelial cells and invasion in the underlying tissue. This subsequently leads to the recruitment of immune cells to the lamina propria and exacerbates the inflammatory state of the tissue, provoking severe and eventually chronic inflammation.^1^ Furthermore, persistent alterations in epithelial cells of IBD patients, including alterations in secretory and absorptive functions, and DNA methylations have been observed.^12–16^

Whether a shift in microbiota is the cause or consequence of IBD remains largely unknown. The implications of host-microbial interactions between epithelial cells and microbiota of different origins, in balance or dysbiosis, needs further elucidation. Furthermore, the role of the host in this response, where epithelial cells of UC patients maintain intrinsic defects, should be unraveled. Therefore, our aim was to evaluate if 1) epithelial cells of UC patients are more sensitive towards microbiota stimulation compared to non-IBD controls and 2) to unravel the effects of microbiota exposure from balanced (healthy volunteer) or microbiota in dysbiosis (UC derived) toward patient specific epithelial cells. In order to do this, an *ex vivo* human organoid-derived Transwell® model was used.

## 2. Results

### 2.1 Patient-derived monolayers maintained patient-specific characteristics

To study interactions between intestinal epithelial cells and microbiota, we used an organoid-derived Transwell® model containing epithelial cells from UC patients or non-IBD controls. To enable the study of active UC, inflammation was (re-) induced in (patient-derived) epithelial monolayers with a previously optimized inflammatory mix.^13^ Twenty-four hours exposure to an inflammatory cytokine mix of 100 ng/mL TNFα, 20 ng/mL IL1β and 1 µg/mL flagellin resulted in upregulation of inflammatory markers and activation of inflammatory pathways, similar to the observed signature previously reported for organoid cultures (Supplementary Figure 1A-C).^13^

We compared organoid-derived monolayers from UC patients and non-IBD controls, in presence or absence of the inflammatory cytokine mix. Untreated UC organoid-derived monolayers maintained disease-specific characteristics, compared to non-IBD controls, with more pronounced changes following inflammatory stimulation. Principal component analysis (PCA) did not reveal a clear separation between UC and non-IBD derived monolayers in both non-inflamed and inflamed conditions, but displayed respectively 31 and 118 differentially expressed genes including IBD related characteristics (e.g. *HLA-G, TNFRSF12A, CLDN1, MUC2*) (Supplementary Figure 2A-D). Thus, patient-specific organoid-derived monolayers enable us to study IBD disease-specific interactions with microbiota, in presence or absence of an inflammatory phenotype, mimicking both UC in an active state and in remission.

### 2.2 Six hours exposure with 3 × 10^8^ microbial cells was optimal for co-culture with epithelial cells

To determine the optimal concentration and duration for co-culture of intestinal epithelial cells with microbiota, different experimental set-ups were evaluated by gene expression analysis and barrier integrity measurements. Concentrations of microbiota were calculated according to the *in vivo* used concentration during FMT and the corresponding epithelial surface area in the Transwell® system.^17^ The organoid-derived epithelial cells in a confluent Transwell® set-up were exposed to increasing concentrations of microbiota of a healthy volunteer (2 x 10^8^, 3 x 10^8^, 5 × 10^8^ and 2 × 10^9^ cells) for 4, 6 and 16 hours.

**Figure 1.**
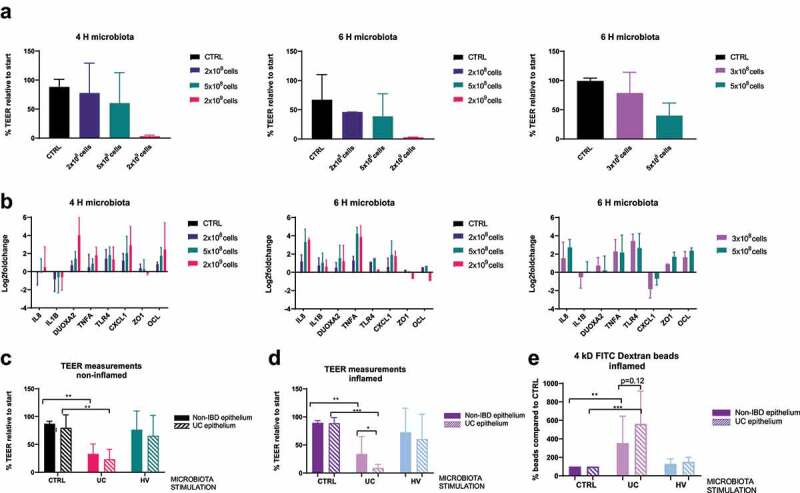
Selection of conditions and barrier integrity measurements. (a-b) TEER measurements (a) and qPCR of inflammatory and tight junctions markers (b) after HV microbiota exposure (2 x 10^8^, 3 x 10^8^, 5 × 10^8^ and 2 × 10^9^ microbial cells) to UC epithelial cells during 4 or 6 hours. (n = 3 for all, except n = 2 for 6 H 2 × 10^9^ cells). (c-d) TEER measurements after 6 hours co-culture with CTRL, UC or HV microbiota (3 x 10^8^ cells) on UC or non-IBD epithelium, without (c) or with inflammatory cytokine mixture stimulation (d). N = 8 for all. (e) 4 kD FITC-dextran (2 mg/mL) permeability assay after 6 hours co-culture with CTRL, UC or HV microbiota (3 x 10^8^ cells) in inflamed epithelial cells. N = 8 for UC epithelium, n = 7 for NON-IBD. TEER is relative percentage compared to measurement start incubation, FITC compared to CTRL condition. CTRL, control; UC, UC microbiota; HV, healthy volunteer microbiota; IF, inflammation; 4 H, 4 hours; 6 H, 6 hours. * p <0.01;** p <0.05; *** p <0.01. Mann-Whitney test (unpaired data) or a Wilcoxon signed rank test (paired data).

Sixteen-hour co-culture experiments resulted in degradation of RNA of epithelial cells (Bioanalyzer, data not shown) and a disturbance of the microbiota community, and were therefore not further executed (Supplementary Figure 3A). A microbiota dose-dependent response of inflammatory markers and alterations in TEER measurements was observed with less pronounced effects at 4 hours (Figure 1a-b, Supplementary Figure 3B). To find an intermediate response between 2 × 10^8^ and 5 × 10^8^ cells, the epithelium was finally exposed to 3 × 10^8^ microbial cells for 6 hours and this set-up was used for follow-up experiments (Figure 1a-b).

Epithelial cells, with or without prior inflammatory stimulation, were co-cultured for 6 hours with vehicle control or microbial cells (3x10^8^ cells) derived from UC patients or a healthy volunteer. After 6 hours, the integrity of the epithelial barrier showed no significant changes in TEER measurements in control conditions (vehicle control, no microbiota exposure), independent of the presence of inflammation (Figure 1c-d).

### 2.3 Exposure to microbiota did not induce a different response between epithelial cells from UC patients and non-IBD controls

In this section, we used transcriptomic analysis and barrier integrity measurements to directly evaluate the difference in host response between epithelial cells from UC patients or non-IBD controls following exposure to microbiota from UC patients or a healthy volunteer. Based on differences in untreated conditions between UC and non-IBD epithelial cells (Graphical abstract A1), we aimed to unravel if epithelial cells of UC patients were more sensitive toward microbiota exposure, compared to non-IBD controls. Monolayers of UC patients and non-IBD controls were exposed to microbiota derived from UC patients with active disease (Graphical abstract A2), or to microbiota of a healthy volunteer (Graphical abstract A3), both in presence or absence of the inflammatory stimuli.

Exposure to UC microbiota induced a clear decrease in epithelial barrier by TEER measurements of both UC and non-IBD epithelial cells, while this response was not observed after exposure of healthy volunteer microbiota (Figure 1c-d). Exposure to UC microbiota resulted in a significantly greater decrease in TEER of epithelial cells of UC patients compared to epithelial cells of non-IBD controls, if performed in presence of an inflammatory cytokine mix (Figure 1d). Although not significant, further analysis with 4kD FITC-dextran showed a trend for decreased barrier integrity in inflamed conditions (Figure 1e).

**Figure 2.**
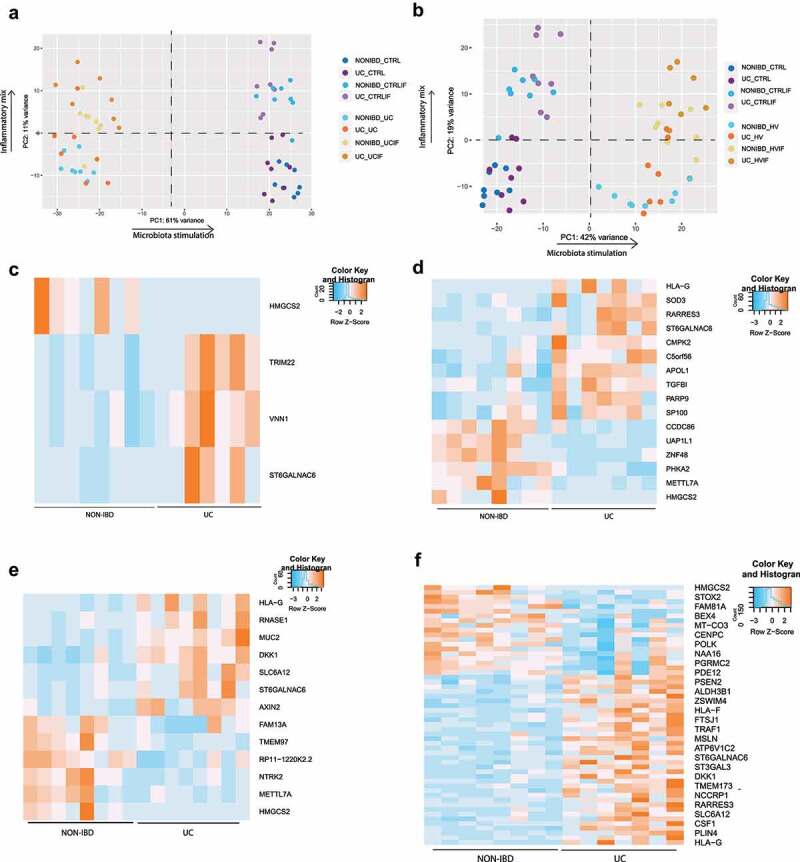
UC and HV microbiota stimulation on epithelial cells of UC patients and non-IBD controls. (a-b) Principal component analysis (PCA) of UC and non-IBD epithelium, with and without UC microbiota exposure (a) or healthy volunteer microbiota exposure (b) and with or without prior inflammatory cytokine mixture stimulation. Lines are drawn to indicate different groups. In all samples together, PCA is driven by microbiota stimulation (PC1), followed by inflammatory stimulation (PC2). (c-f) Heatmap showing expression levels of significant DEG (FDR <0.05) between UC and non-IBD epithelial cells in non-inflamed (c) and inflamed (d) conditions after UC microbiota exposure or between UC and non-IBD epithelial cells in non-inflamed (D) and inflamed (e) conditions after HV microbiota exposure. Labels are as following: tissue_exposure; CTRL: control; HV, healthy volunteer; IF, inflammation; NON-IBD, non-IBD control; UC: ulcerative colitis.

For transcriptomic analysis, PCA was driven by the origin of microbial treatment, followed by stimulation with the inflammatory mixture. However, no clear differential response between epithelial cells from UC patients or non-IBD controls exposed to microbiota was seen, whether stimulated with an inflammatory cytokine mix or not, both following UC or healthy volunteer microbiota stimulation (Figure 2a-b).

In the non-inflamed setting, exposure to UC microbiota resulted in the identification of 4 differentially expressed genes (FDR <0.05) between UC and non-IBD epithelial cells (Figure 2c). In inflamed conditions, treatment with UC microbiota caused 16 differentially expressed genes including *PARP9, TGFBI* and *CMPK2* (Figure 2d). Seven of these 16 genes were significantly differentially expressed in unexposed conditions, while nine were specifically related to microbiota exposure. These genes were linked to mitochondrial pathways (*HMGCS2, CMPK2*) and repair of DNA damage (*PARP9*). However, aside from this limited number of genes, no clear distinct transcriptomic or barrier integrity response could be identified.

Exposure to microbiota from the healthy volunteer did neither result in a major differential response between UC and non-IBD epithelial cells (Figure 2b). In non-inflamed settings, only 13 differentially expressed genes were detected (Figure 2e). For inflamed settings, this increased up to 55 differentially expressed genes including *MUC2, RNASE1, TGFBI* (Figure 2f), but still remarkably lower than the control condition (no microbiota exposure) in which respectively 31 (non-inflamed) and 118 (inflamed) differentially expressed genes were found. Hence, also for healthy volunteer microbiota, transcriptional changes were driven by microbial exposure and not epithelial origin.

### 2.4 Co-expression modules were mainly associated with characteristics of untreated epithelial cells

To complement differential gene expression analysis, we used weighted gene co-expression network analysis (WGCNA) to assess clusters of genes with similar expression patterns. Comparison of epithelial cells from UC and non-IBD controls in both inflamed and non-inflamed conditions, resulted in the identification of 11 co-expression clusters ranging in size from 77 to 2015 genes. In absence of microbiota, three clusters significantly correlated with UC non-inflamed epithelium as compared to non-IBD epithelium; of which one cluster significantly correlated with inflamed UC epithelium (turquoise cluster). The significant clusters identified in absence of microbiota were involved in tight junction and integrin signaling, and DNA damage response (BRCA1). The pink cluster was linked to the role of PKR in interferon induction and antiviral responses. In contrast, upon exposure to microbiota from the healthy volunteer only one significant correlation (pink cluster) was identified in inflamed conditions (Supplementary Figure 4). Again, the highest number of involved clusters was detected in the control condition, indicating that differences between UC and non-IBD epithelial cells were mainly driven by baseline characteristics and not induced by exposure to microbiota of UC patients or the healthy volunteer.

### 2.5 UC microbiota induced a strong response in inflamed epithelial cells, that was not present following HV microbiota exposure

As a second research question, we aimed to analyze host-microbial interactions by studying the effect of different types of microbiota exposure (patient derived vs. healthy volunteer derived) toward epithelial cells (Graphical abstract B). As we did not observe a different transcriptional response between UC and non-IBD epithelial cells, we first analyzed the response in inflamed UC epithelial cells, to mimic active disease. Next, we also confirmed expression of key genes in non-inflamed and non-IBD epithelial cells.

**Figure 3.**
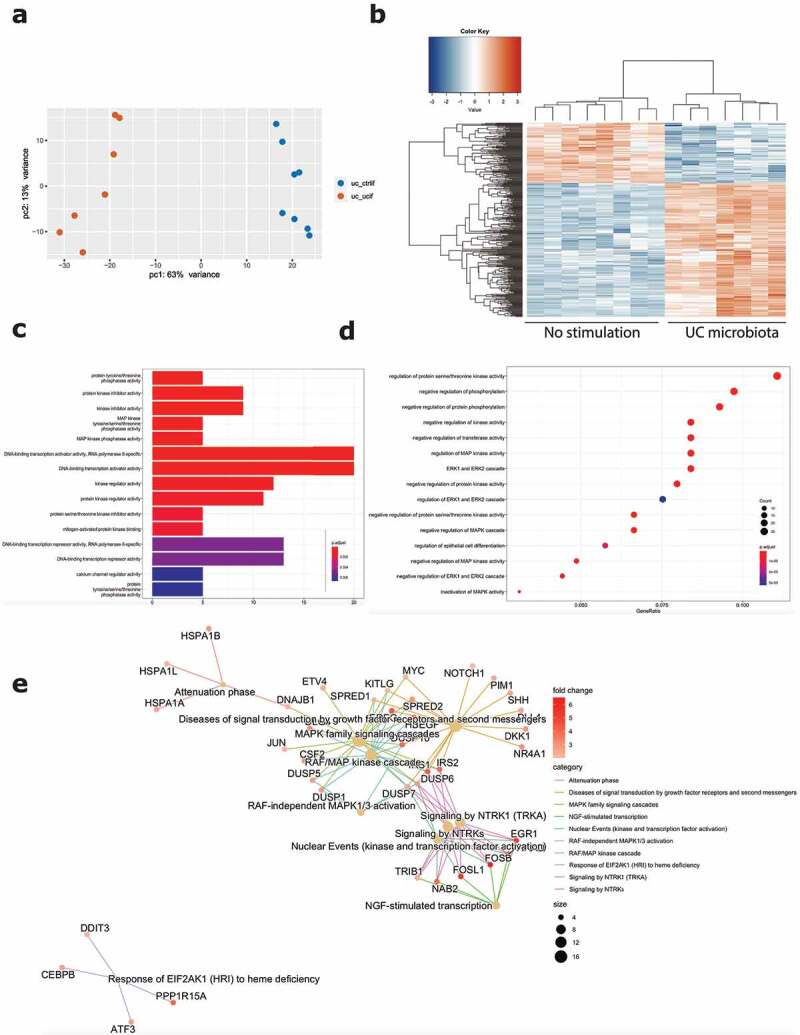
Effect of UC microbiota on inflamed UC epithelial cells. (a) PCA displaying separate clustering between inflamed UC epithelial cells without and with exposure to UC microbiota. (b) Heatmap showing significant differentially expressed up- and downregulated genes (FDR ≤ 0.01 and absolute log2 fold change ≥2) between inflamed UC epithelial cells without and with UC microbiota exposure. (c-e) Functional enrichment analysis showing significant upregulated pathways between inflamed UC epithelial cells without and with exposure to UC microbiota by (c) Gene ontology molecular functions top 15 (d) Gene ontology biological processes top 15 (e) Reactome analysis. Labels are as following: tissue_exposure; CTRL: control; IF, inflammation; UC: ulcerative colitis.

For the first comparison, inflamed epithelial cells from UC patients were exposed to microbiota derived from UC patients with active disease, compared to inflammatory stimulation only (Graphical abstract B1). Barrier integrity measurements (TEER and FITC) showed a strong decrease following stimulation with UC microbiota (Figure 1d-e). Because of the high number of DEGs (6634 DEG, FDR ≤0.05), a more stringent cutoff (log2FC = 2, FDR ≤ 0.01) for downstream transcriptomic analysis was applied. This way, exposure of UC epithelial cells toward UC microbiota resulted in 354 DEG, including 110 downregulated and 244 upregulated genes, compared to the control condition (Figure 3a-b). Top upregulated genes included *EGR1, FOSL1, FOSB* and GEM and top downregulated genes comprised *CHP2, SLC26A2, GPR128* and *TM4SF20* (Supplementary Figure 5A).

**Figure 4.**
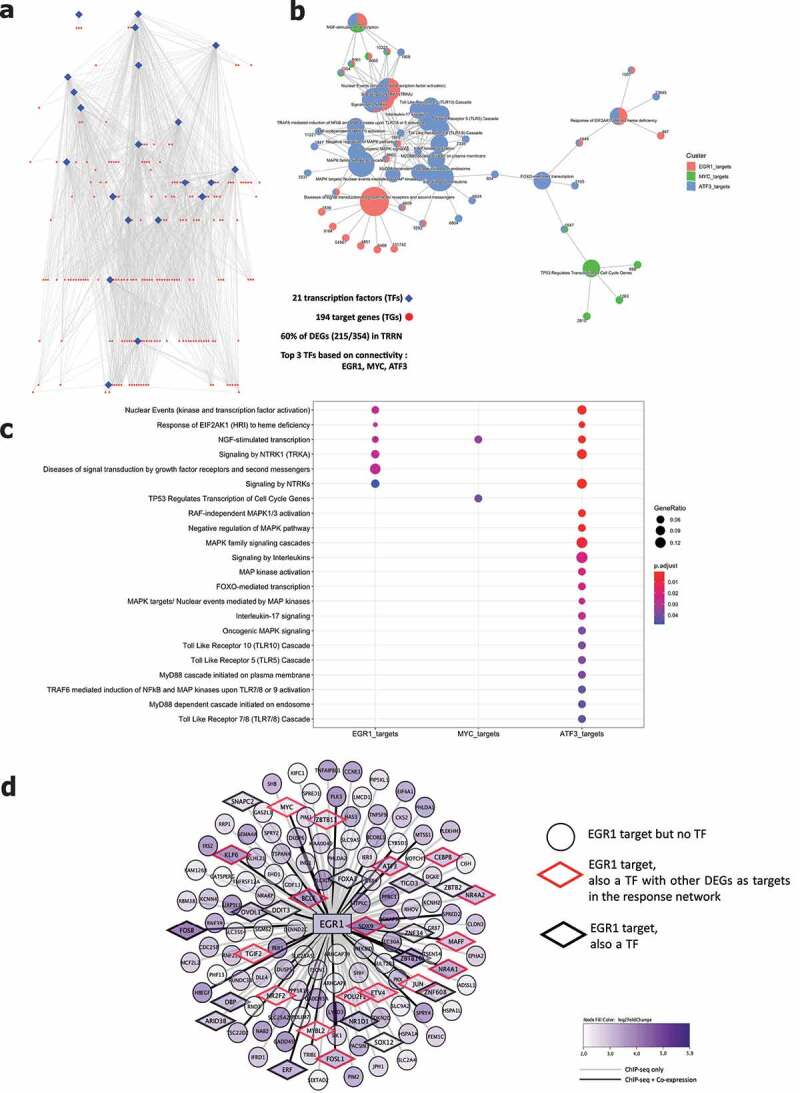
Transcriptional regulatory response network following UC microbiota stimulation. (a) Visualization of the transcriptional regulatory response network (TRRN) of UC epithelial cells following UC microbiota exposure. In the network, 21 transcription factors and 194 target genes were identified. Sixty percent of the overall differentially expressed genes are involved in this network. The top 3 transcription factors based on connectivity in the network are EGR1, MYC and ATF3. (b-c) Visualization of functions and pathways in which the top 3 transcription factors are involved. (d) EGR1 acts as a master upregulated by upregulating <101 genes and 32 other transcription factors following UC microbiota exposure on UC epithelial cells. Seventeen of the 32 EGR1 targeted transcription factors also regulated other DEGs in the response network.

Analysis by gene ontology molecular functions demonstrated activation of molecular mechanisms including kinase inhibitor/regulator activities (e.g. *CKS2, SPRY4, CCNE1*) and DNA-binding transcription activators (e.g. *EGR1, MYC, FOSB, KLF6*) (Figure 3c). Gene ontology for biological processes identified negative regulation of multiple protein kinase activities (e.g. *DKK1, IRS2, SFN*) and enrichment of the MAPK and ERK1 and ERK2 cascade (e.g. *ATF3, MYC, CDC6*) (Figure 3d). Reactome analysis showed activation of multiple processes involved in the RAF/MAP pathway (e.g. *DUSP1, IRS1-2, JUN*) signaling by NTRKs (e.g. *FOSB, FOSL1, IRS1-2*) and nuclear events (e.g. *FOSB, FOSL1*) (Figure 3e). Evaluation of transcriptional regulators by transcriptional regulatory response network (TRRN) analysis showed 21 involved transcription factors (TFs) modulating expression of 194 target genes (TGs) (Figure 4a). The three major involved TFs (based on connectivity) include EGR1, MYC and ATF3 (Figure 4b-c). Based on the upregulation of many target genes (>100) including several other transcription factors, EGR1 appears to act as a master regulator mediating the influence of UC microbiota stimulation on epithelial cells (Figure 4d).

**Figure 5.**
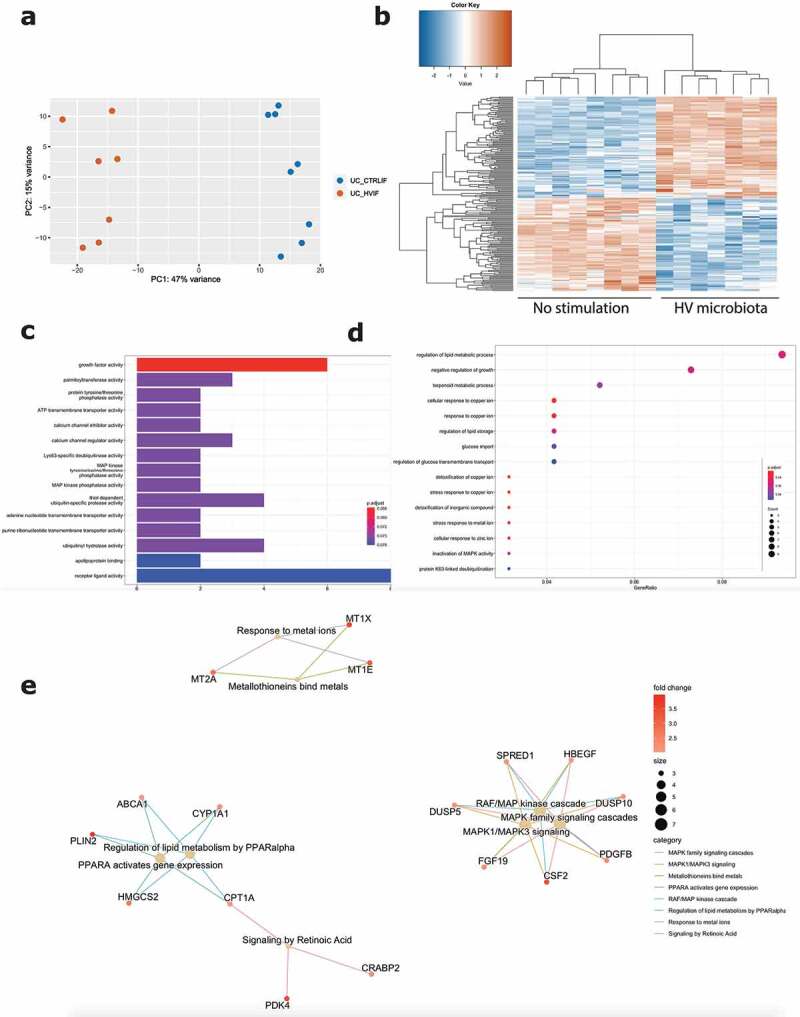
Effect of healthy volunteer microbiota on inflamed UC epithelial cells. (a) PCA displaying separate clustering between inflamed UC epithelial cells without and with exposure to HV microbiota. (b) Heatmap showing significant differentially expressed up- and downregulated genes (FDR ≤ 0.01 and absolute log2 fold change ≥2) between inflamed UC epithelial cells without and with exposure to healthy volunteer microbiota. (c-e) Functional enrichment analysis showing significant upregulated pathways between inflamed UC epithelial cells without and with exposure to healthy volunteer microbiota by (c) Gene ontology molecular functions top 15 (d) Gene ontology biological processes top 15 (e) Reactome analysis. Labels are as following: tissue_exposure; CTRL: control; IF, inflammation; HV, healthy volunteer; UC: ulcerative colitis.

Next, we focused on the effect of exposure to microbiota of a healthy volunteer toward inflamed epithelial cells from UC patients (Graphical abstract B2). Barrier integrity measurements showed no significant decrease following exposure to healthy volunteer microbiota, compared to the control condition without microbiota (Figure 1d-e). Also transcriptomic analysis demonstrated less pronounced effects with only 196 DEGs (log2FC = 2, FDR ≤0.01; 6093 DEGs with FDR <0.05), including 102 upregulated and 94 downregulated genes (Figure 5a-b). The top upregulated genes included *CSF2, MCF2L2, BCORL1* and *ANKRD22*, while downregulated genes included *IFIT1, CXCL10, SNPH* and *SEPP1* (Supplementary Figure 5B). While UC microbiota top upregulated genes were UC microbiota specific (Supplementary Figure 5C), top upregulated genes following healthy volunteer microbiota were also observed following UC microbiota exposure and thus not microbiota specific (Supplementary Figure 5D). Moreover, the number of identified pathways was remarkably lower. Gene ontology showed activation of molecular mechanisms including growth factor activity (e.g. *CFS2, FGF19)*, calcium channels and activations of several transmembrane transporters (e.g. *PACSIN3, SLC25A42*) (Figure 5c). Gene ontology for biological processes illustrated mainly a response toward copper and zinc ions (e.g. *MT1E, MT2A*), but also glucose import and regulation of lipid storage (e.g. *PLIN2, ABCA1*) (Figure 5d). In addition, Reactome analysis displayed limited pathways including the RAF/MAP pathway and response to metal ions (e.g. *FGF19, CSF2*) (Figure 5e). TRRN analysis did not reveal any meaningful results to infer master transcriptional regulators following exposure to microbiota from the healthy volunteer (data not shown).

### 2.6 Exposure toward microbiota from patients with UC induced a more detrimental effect compared to microbiota of a healthy volunteer

Next, we directly compared the differences between exposure to UC microbiota or microbiota of a healthy volunteer (Graphical abstract B3). Comparison of DEGs in both conditions (both compared to the control condition) showed shared and unique genes to each set. From the DEGs in both conditions (CTRL vs. UC microbiota and CTRL vs. healthy volunteer microbiota), 46 of the upregulated genes and 33 of the downregulated genes were dysregulated in both conditions, while the remaining genes were unique for the type of microbiota used (Supplementary Figure 6A). Reactome pathway analysis showed unique pathways for UC microbiota (e.g. Nuclear events, Attenuation phase) or the healthy volunteer microbiota (e.g. *PPARA* activated gene expression, regulation of lipid metabolism), as well as shared pathways (Supplementary Figure 6B). Further analysis by additional databases demonstrated activation of the JAK-STAT pathway, GPCR signaling following UC microbiota but not after healthy volunteer exposure (Supplementary Figure 6C).

**Figure 6.**
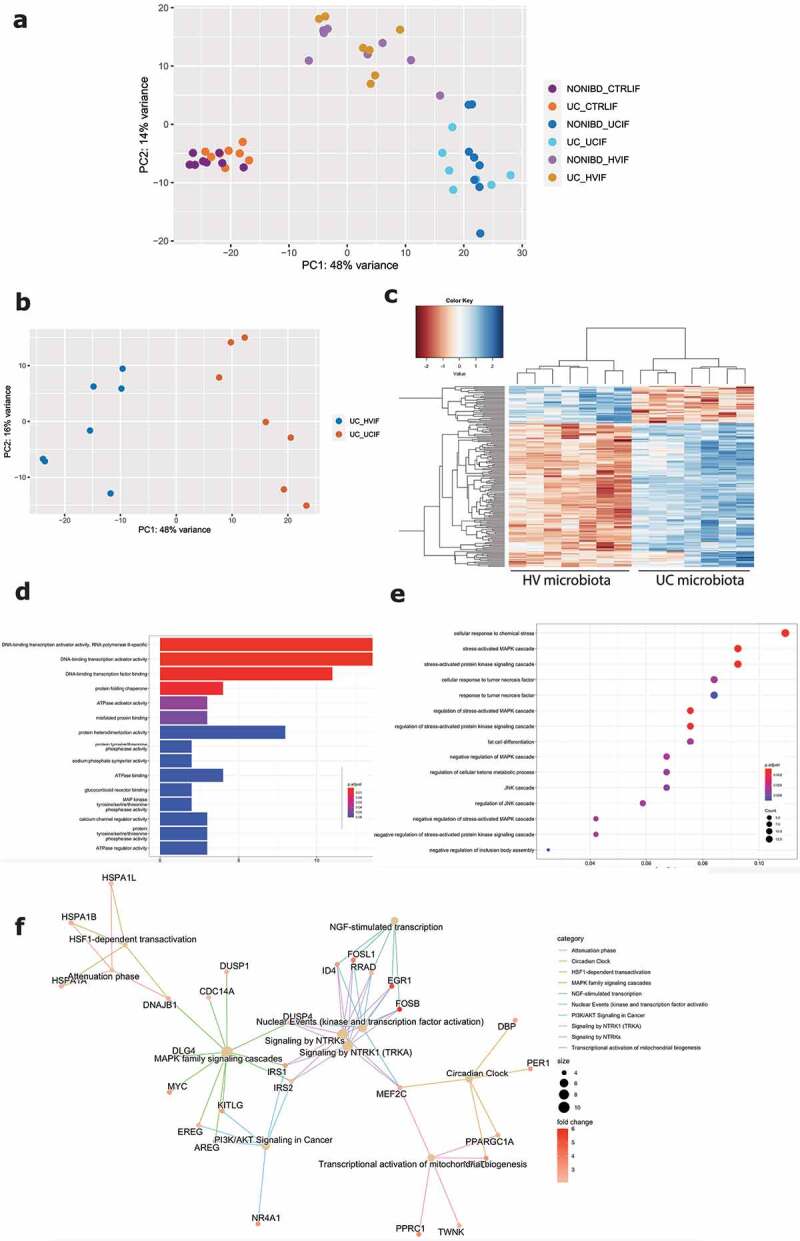
Comparison of healthy volunteer microbiota and UC microbiota on epithelial cells. (a) PCA showing clustering driven by microbial treatment, with HV microbiota located between no treatment and UC microbiota. (b) PCA displaying separate clustering between inflamed UC epithelial cells with UC and HV microbiota exposure. (c) Heatmap showing significant differentially expressed up- and downregulated genes (FDR ≤ 0.01 and absolute log2 fold change ≥2) between inflamed UC epithelial cells with UC and HV microbiota exposure. (c-e) Functional enrichment analysis showing significant upregulated pathways between inflamed UC epithelial cells with UC and HV microbiota by (d) Gene ontology molecular functions top 15 (e) Gene ontology biological processes top 15 (f) Reactome analysis. Labels are as following: tissue_exposure; CTRL: control; HV, healthy volunteer; IF, inflammation; NON-IBD, non-IBD control; UC: ulcerative colitis.

PCA including the control without microbiota exposure, UC microbiota and healthy volunteer microbiota samples showed clustering based on microbial treatment, with healthy volunteer microbiota located between the two extremes (Figure 6a). The direct comparison between exposure to UC or healthy volunteer derived microbiota on UC epithelial cells identified 161 DEG including 128 up- and 33 downregulated genes (Figure 6b-c). Molecular functions showed higher activation of DNA binding transcription activation (e.g. *MYC, NR4A2*), misfolded protein binding (e.g. *HSPA1A-B*) and ATPase activity after exposure toward UC microbiota (Figure 6d). Biological processes gene ontology analysis included multiple cell responses involved in (chemical) stress, tumor necrosis factor, and the MAPK and JNK cascade (e.g. *DACT1, CCL20, MYC, CCN2*), that were after UC microbiota (Figure 6e). Finally, Reactome analysis showed increased activation of PI3K/AKT signaling, the attenuation phase and activation of mitochondrial biogenesis (e.g. *AREG, FOSB, IRS1-2*) (figure 6f), following UC microbiota exposure. Individual inflammatory markers (IL-8, TNF-α, CCL20) were higher expressed after UC microbiota exposure, compared to microbiota from a healthy volunteer (Supplementary Figure 7). We confirmed expression levels of key genes in (non-) inflamed epithelial cells from non-IBD controls and non-inflamed UC epithelial cells, and showed that PCA was driven by microbial treatment, followed by inflammatory stimulation, and not by epithelial origin (Supplementary Figure 8A-B).

### 2.7 The microbial composition is not influenced by the epithelial cell type

**Figure 7.**
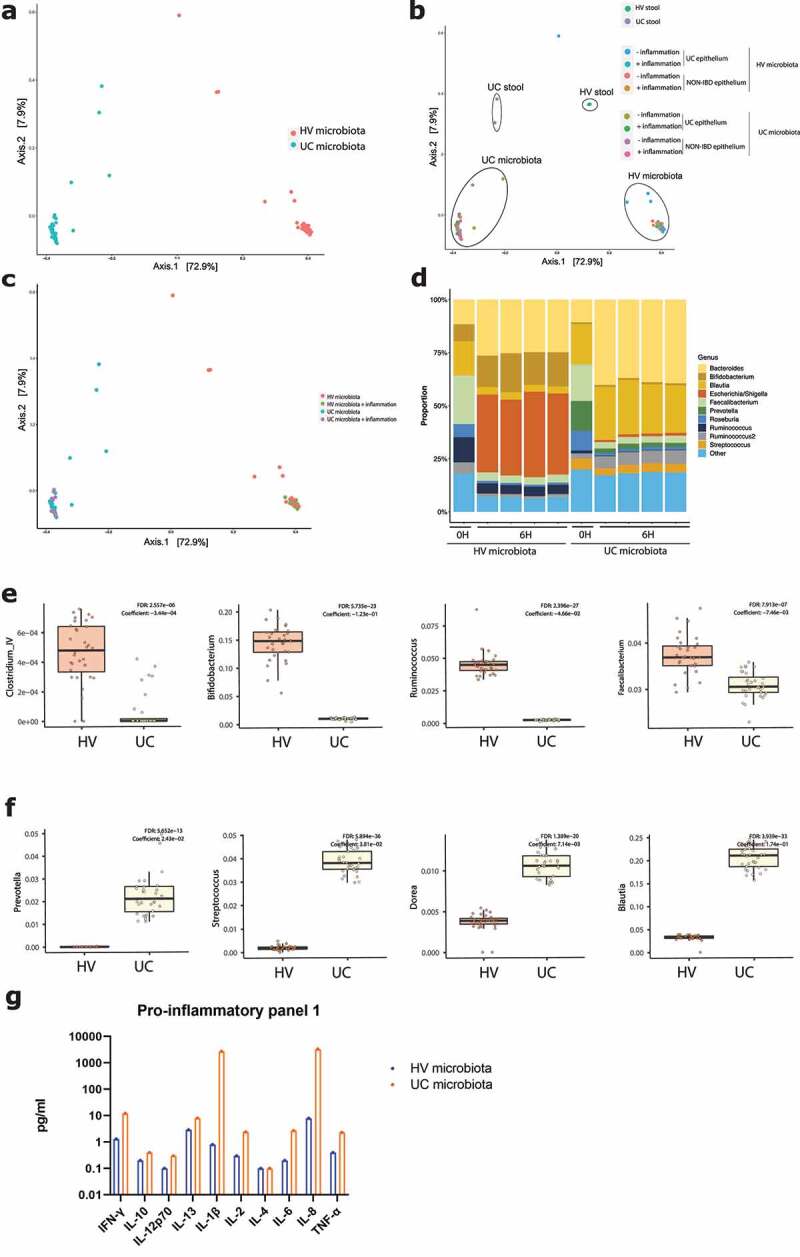
16S rRNA sequencing of microbiota samples before and after co-culture. (a) Principal coordinates analysis (PCoA) showing separate clustering based on microbiota origin (UC, n = 34 and HV microbiota, n = 34). (b) PCoA showing separate clustering of stool samples (UC and HV microbiota, both n = 2) and microbiota after 6 H co-culture (both n = 32). Circles drawn to indicate different treatment groups. (c) PCoA showing no effect of inflammation on microbiota composition. HV ± inflammation, both n = 16 (d) Composition of microbiota samples exposure at genus level. Samples after 6 hours from left to right, NON-IBD epithelium without and with inflammation, UC epithelium without and with inflammation. Stool sample n = 2, after 6 hours n = 8 for all samples. (e) After six hours, microbiota samples of the healthy volunteer show higher levels of *Clostridium IV, Bifidobacterium, Ruminococcus* and *Faecalibacterium*. (f) After six hours, microbiota samples of the UC donors show higher levels of *Prevotella, Streptococcus, Dorea* and *Blautia*. (g) Analysis of inflammatory markers of microbiota samples from the UC donors and HV prior to epithelial cell exposure by V-PLEX Pro-inflammatory Panel 1. HV, healthy volunteer; IF, inflammation; NON-IBD, non-IBD control; UC: ulcerative colitis; 0 H: 0 hours; 6 H: 6 hours.

To analyze the microbial community, 16S rRNA sequencing was performed on microbiota samples, before and six hours after co-culture with epithelial cells. Principal coordinates analysis (PCoA) on individual microbial samples was driven by microbial treatment, and not by the co-cultured epithelial cell type (Figure 7A-B). Neither did inflammation affect the composition of microbial samples (Figure 7C). The composition shifted during the 6 hours of exposure, phyla of interest levels remained high after 6 hours (Figure 7D). We observed higher levels of *Ruminococcus, Bifidobacteria, Faecalibacterium* and *Clostridium IV* after 6 hours co-culture in the microbiota from the healthy volunteer, compared to the UC microbiota (Figure 7E). Accordingly, higher levels of *Streptococcus, Blautia, Dorea* and *Prevotella* were found in the microbial mix from UC patients after 6 hours (Figure 7F). Protein analysis of pro-inflammatory markers showed higher expression of IL-8 and IL-1β in microbiota samples of UC patients, compared to the healthy volunteer sample (Figure 7G).

## 3. Discussion

Epithelial cells from UC patients possess intrinsic defects, both in presence or absence of inflammatory stimuli.^12,13^ In this study, we examined if the epithelium from UC patients is more sensitive toward microbiota stimulation, compared to the epithelium from non-IBD controls. We also studied if different types of microbiota (balanced vs. dysbiosis) exert distinct effects on epithelial cells. Using organoid-derived monolayer cultures, no pronounced difference between the epithelium from UC patients and non-IBD controls after exposure to microbiota (balanced or dysbiosis) was seen. In contrast, compared to stimulation with microbiota from a healthy volunteer, exposure to microbiota from UC patients induced a damaged and stress induced phenotype in inflamed epithelial cells from UC patients. Expression of key genes was also confirmed in (non-) inflamed non-IBD epithelial cells.

FMT is proposed as a novel treatment strategy for patients with UC and several randomized control trials showed promising effects.^18–20^ To further study mechanisms by which FMT might work, we applied microbiota samples with similar concentrations according to the treated surface. The aerobic setting, required for epithelial survival, limited the time-span of co-culture to six hours but was sufficient to induce major gene expression changes. The current organoid model is associated with its limitations and, for future experiments, models with both aerobic and anoxic chambers, and other cell types should be implement. Still, the combination of patient specific epithelial cells and microbial samples in the current set-up is superior to the use of traditional cell culture models.^21^ Relative levels of several genera of interest were higher in healthy volunteer samples (e.g. *Clostridium IV, Bifidobacterium, Ruminococcus, Faecalibacterium)*, indicating that also after six hours fecal donor characteristics were conserved. Following six hours co-culture, a relative higher percentage of the facultative anaerobe commensal, *Escherichia (E.) coli* was observed in healthy volunteer samples. *E. coli* can survive in aerobic conditions with limited nutrients and can easily adapt to the used culture conditions.^22,23^ Also in another study that used a co-culture model including an anaerobe compartment, the initial microbial community showed changes over time.^24^

The inclusion of quantitative microbiome measurements (in addition to relative comparisons), emerged as a major change in the microbiome field.^25^ Microbiota in dysbiosis from UC patients is associated with lower absolute bacterial cell counts and a decrease in biodiversity and richness.^8–10^ Due to a much lower absolute microbial cell numbers in UC patients, a higher weight and volume of the original sample was needed to reach the required bacterial cell amount and corrected by equalizing fecal samples in the same volume of diluent. One factor we could hereby not take into account was the absolute numbers and species of fungi, viruses but also fibers and metabolites that can possibly contribute to the read-out. In addition, we found higher levels of IL-8 and IL-1β in the microbiota samples of UC patients, which might possibly contribute to an inflammatory response, although inflammation was previously induced. Furthermore, we analyzed response to one UC microbiota mix thus it should be investigated if this response is triggered by presence of specific phyla in UC microbiota that can potentially be targeted. Further experiments to unravel the role of specific bacterial strains, including mucus-associated bacteria should be performed. Complementary, inflammation may be patient specific and our inflammatory mix might not be representative for every patient. Also, mucus production is observed in these *ex vivo* monolayers but remains lower compared to the *in vivo* situation.^26^ Of note, in UC patients with active disease, this mucus layer is often disrupted leading to contact between epithelial cells and the microbiota.^27^

We acknowledge that some epithelial characteristics might fade over time during organoid cultures, but an IBD linked gene expression profile was maintained in the model. We observed a larger difference between UC and non-IBD derived organoids and, more pronounced UC specific phenotype, following inflammatory stimulation, and hypothesized that epithelial cells from UC patients were more sensitive toward microbiota stimulation. However, we could not identify a different response between both types of epithelial cells following microbiota stimulation. Conceivably, microbiota exposure, especially from UC patients, overpowers the baseline differences between epithelial cells and is a more important trigger compared to the host. However, it cannot be excluded that, in the presence of other components (e.g. patient specific immune cells), a different response would be observed.

Next, we evaluated direct effects of different types of microbiota on inflamed epithelial cells from UC patients. Exposure to microbiota from UC patients was sufficient to induce damage to the epithelial barrier and activate several stress pathways. Diverse pathways were dysregulated including signaling by NTRK and major activation of RAF/MAP, MAPK signaling and the JAK/STAT pathway following UC microbiota exposure. The top upregulated genes include FOSL1 and FOSB, both belong to the activator protein-1 (AP-1) family, modulated by the MAPK pathway.^28^ The MAPK pathway activates through phosphorylation specific cellular responses to internal and external stress signals, including phosphorylation of pro-inflammatory proteins, and dysregulation of this pathway is linked to multiple diseases including IBD and tumorigenesis.^29–32^ Activation of protein kinase inhibitors and regulators was observed after UC microbiota, and is linked to the IBD pathogenesis and microbial processes.^31,33^ Also, the JAK/STAT pathway is an important therapeutic target in IBD.^34^ Several of the detected genes are involved in the regulation of intestinal cell apoptosis (*ATF3*^35^), intestinal barrier function (*SPRY4-IT1*^36^), inflammation and wound repair (*DKK1^37^*).

Upon exposure to UC microbiota, EGR1 was identified as master regulator by TRRN analysis and is a transcription factor activated through MAPK signaling, involved in tissue injury, immune responses and modulation of TNF-α.^38–41^ Dong et al. found increased gene expression and activation levels of EGR1 in biopsies from UC patients, compared to healthy controls.^42^ Also MYC, another top three transcription factors, was found at higher levels in biopsies from patients with IBD by Macpherson et al. and linked to altered cell cycle control due to inflammatory processes.^43^

By contrast, exposure to microbiota from a preselected healthy volunteer had milder effects. The top upregulated genes after exposure to healthy volunteer microbiota were also upregulated after UC microbiota, indicating that this is a general microbiota response and not specific to the type of microbiota.

The direct comparison between UC and healthy volunteer microbiota exposure demonstrated increased activation of stress cascades, indicating a higher stress level in epithelial cells when exposed to UC microbiota. Pathways involved in IL-4, −13, −17 signaling and the JNK pathway, a subgroup of MAP kinases, were upregulated. JNK1/2 kinase activity was found to be enriched in the colon of active IBD patients and represents a potential treatment target.^32,44^

Together, our results show that the type of microbiota, and not the origin of epithelial cells, drives epithelial cell response. Expression of key markers confirmed our findings in non-IBD epithelial cells, and UC epithelial cells without inflammatory stimulation. Although transplantation of microbiota from colitis to wild-type mice,^5^ or restoration of the fecal stream in patients is sufficient to induce an inflammatory phenotype,^6,7^ we show that even in the absence of immune cells, exposure of epithelial cells to UC microbiota is sufficient to activate important stress and injury associated pathways, as well as IBD linked genes. Our results suggest that only targeting the immune cell infiltrate may not be sufficient to resolve inflammation, as contact between epithelial cells and microbiota is sufficient to induce stress and injury cascades.

Further research on microbiota modulation, either by FMT, multifunctional bacteria consortia, or other approaches including diets, should be performed to supplement classic treatment option and remove the constant trigger of dysbiosis. Combining conventional therapies (resolving inflammation), and restoration of microbiota will possibly lead to a prolonged maintenance of a balanced microbiota and overall homeostasis. How this should be reached remains one of the major IBD-related research questions in the coming years.

## 4. Material and methods

### 4.1 Patient biopsy collection

Eight non-IBD controls and eight patients diagnosed with active UC, classified as an endoscopic Mayo subscore of ≥2 but with an accessible margin between macroscopically inflamed and non-inflamed mucosa, were included at the University Hospitals Leuven (Leuven, Belgium). Fresh mucosal biopsies from non-inflamed colon segments were collected during routine endoscopy. Non-IBD controls underwent endoscopic evaluation for polyp screening or nonspecific gastrointestinal complaints, but did not have any macroscopic abnormalities. Baseline characteristics of UC patients, non-IBD controls and organoids are given in Supplementary table 1. The diagnosis of UC was based on ECCO-ESGAR guidelines for IBD.^45^ Fresh fecal samples were collected from two patients within this cohort, as well as one healthy volunteer (selection criteria described below). The study was approved by the ethical committee of the University Hospitals Leuven (S53684 and S59525), and all patients gave written informed consent prior to sample collection. All authors had access to the study data and had reviewed and approved the final manuscript.

### 4.2 Processing of fecal samples

Fresh samples of a preselected healthy volunteer and two UC patients were stored at 4°C including an anaerobic patch and processed within five hours in a Whitley A35 Anaerobic Workstation. All weighted samples were dissolved in 0.9% saline, mixed with a magnetic stirrer, filtered using a Minisart syringe filter (5 µm). Next, the cells were counted using flow cytometry (BD AccuriTM C6) and diluted until 10^10^ bacterial cells/mL. The sample was supplemented with 10% glycerol and frozen at −80°C until further use.

The asymptomatic healthy volunteer was selected on high microbial load, and presence of phyla of interest.^46^ Exclusion criteria included active smoking, antibiotic use in the past three months, any medical history, increased inflammation markers, the presence of enteric pathogens and familial history of IBD. The applied UC microbiota was a mix of three fecal samples from two distinct patients with active disease (Supplementary table 1) and classified as Bristol 6–7, linked to low bacterial abundance.^25,47^ Three UC samples were combined into one mix, to prevent a specific patient related species specific response, and an absolute higher weight of UC samples was required to obtain the same cell count as for the healthy volunteer. For optimization experiments (concentration and duration co-culture), another pre-screened healthy volunteer was used.

### 4.3 Crypt isolation, organoid and Transwell® culturing

Crypts were isolated, expanded as organoids and cultured as Transwell® cultures as described before.^13,48^ Further information can be found in the supplementary material and methods.

### 4.4 Co-culture microbial and epithelial cells

Inflammation was re-induced in confluent Transwell® cultures, 24 hours prior to microbiota co-culture, if applicable. Transwells® were exposed to 100 ng/mL TNFα (Invivogen, San Diego, California), 20 ng/mL IL1β (Peprotech, London, UK) and 1 µg/mL flagellin (tlrl-stfla, Invivogen), similar to previous organoid experiments.^13^ Twenty-four hours later, cultures were washed three times with BM without antibiotics before the microbiota suspension (apical compartment) and – if applicable – the inflammatory cytokine mix (basolateral compartment) were added for six hours at 37°C and 5% CO_2_. Cells were stimulated with 3 × 10^8^ microbial cells or vehicle control (70 µl 0.9% NaCl and 230 µl BM without antibiotics). After six hours co-culture, TEER was remeasured. All cultures were washed three times with BM including penicillin and streptomycin. Cultures were used either for 1) hematoxylin and eosin staining 2) FITC-dextran (4 kD) permeability measurements, or 3) RNA extraction. These procedures are described in supplementary material and methods.

### 4.5 RNA sequencing

RNA libraries were prepared with TruSeq Stranded mRNA library kit (Illumina, California, USA) and sequenced by Illumina HiSeq4000 (Illumina), with an average sequencing depth of 23 million reads per sample. Three samples had to be excluded due to low quality reads (Supplementary table 2). RNA-seq data have been deposited in the ArrayExpress database at EMBL-EBI (www.ebi.ac.uk/arrayexpress) under accession number E-MTAB-10832.

### 4.6 Differential expression and functional enrichment analysis

Alignment of raw RNA-sequencing data to the human reference genome hg19 was performed through HISAT2, absolute counts were generated using HTSeq count v0.5.3p3. Only protein coding genes (as per GENCODE annotation – release 30 GRCh38.p12),^49^ were considered. Only genes with greater than 10 counts in at least 80% of samples corresponding to the compared conditions were considered for further analysis. Post-normalization, differential expression analysis was performed using DESeq2.^50^

Genes with FDR ≤ 0.05 (epithelial cell comparison) were considered to be differentially expressed genes (DEG). For microbiota comparison, a more stringent cutoff (FDR ≤ 0.01 and absolute log2 fold change ≥2) was applied due to the high number of DEG. Pathway analysis was performed in Ingenuity Pathway Analysis (QIAGEN Inc., https://www.qiagenbioinformatics.com/products/ingenuity-pathway-analysis,^51^ on differentially expressed genes (FDR <0.05). Additionally, functional enrichment analysis of DEGs was performed using clusterProfiler^52^ and ReactomePA^53^ packages. Enrichment events (FDR ≤ 0.1) corresponding to gene sets with at least 10 genes were considered statistically significant. WGCNA, additional functional enrichment analysis and Regulatory network construction are described in the supplementary material and methods.

## 4.7 16S rRNA sequencing

16S rRNA sequencing of microbial samples was performed before and after co-culture with epithelial cells and is described in the supplementary material and methods.

### 4.8 Mesoscale analysis

Levels of pro-inflammatory markers were measured in the microbiota samples using the V-PLEX Pro-inflammatory Panel 1 (human) Kit (Meso Scale Discovery, Rockville, MD) according to the manufacturer’s protocol. A 2-fold dilution of the applied microbiota samples (3x10^8^ cells in 70 µl) was measured on the Meso Scale Discovery (MSD) QuickPlex SQ120 M instrument and evaluated on the MSD software platform.

### 4.9 Statistics

Statistical analysis was performed with GraphPad Prism 9 software (San Diego, California, USA) and RStudio 4.0.4 (The R foundation, Vienna, Austria). All displayed significance values for RNA sequencing are adjusted p-values (FDR value) unless stated otherwise. TEER measurements were analyzed by a Mann-Whitney test (unpaired data) or a Wilcoxon signed rank test (paired data). Continuous variables on graphs were expressed as median and interquartile range (IQR).

## Supplementary Material

Supplemental Material

## Abbreviations

ADJ p value Adjusted p-value; BM Basal medium; CTRL Control; CXCL Chemokine (C-X-C motif) ligand; DEG Differentially expressed genes; FDR False discovery rate; FITC Fluorescein isothiocyanate; FMT Faecal microbiota transplantation; HM Human expansion medium; INF Inflamed; IQR Interquartile range; ISC Intestinal stem cells; NA Not applicable; NON-INF Non-inflamed; NS Not significant; PCA Principal component analysis; PCoA Principal coordinates analysis; RPS14 Ribosomal protein S14; RTqPCR Real-time quantitative polymerase chain reaction; TEER Transepithelial electrical resistance; TNF Tumor necrosis factor; TRRN Transcriptional regulatory response network; WGNCA Weighted gene co-expression network analysis; 16S rRNA 16S ribosomal RNA.

## Transcript profiling

EMBL-EBI ArrayExpress database (www.ebi.ac.uk/arrayexpress) under accession number E-MTAB-10832.

## Data transparency

RNA sequencing data is publicly available at the EMBL-EBI ArrayExpress database (www.ebi.ac.uk/arrayexpress) under accession number E-MTAB-10832.
