# Supplementary material for: Microbiota, not host origin drives ex vivo intestinal epithelial responses
Source: Gut Microbes. 2022 Jun 26;14(1):2089003. doi: 10.1080/19490976.2022.2089003 (PMC9235885; doi:10.1080/19490976.2022.2089003)
Supplement: Supplemental Material [file KGMI_A_2089003_SM7781.pdf]

Supplementary data

Microbiota, not host origin drives *ex vivo* intestinal epithelial response

Arnauts Kaline [1,2], Sudhakar Padhmanand [1], Verstockt Sare [1], Lapierre Cynthia [1], Potche Selina [1], Caenepeel Clara [1,3], Verstockt Bram [1,3], Raes Jeroen [4], Vermeire Séverine[1,3], Sabino João [1,3], Verfaillie Catherine [2], Ferrante Marc [1,3]

[1]Department of Chronic Diseases, Metabolism and Ageing (CHROMETA), Translational Research Center for Gastrointestinal Disorders (TARGID), KU Leuven, Leuven, Belgium

[2]Department of Development and Regeneration, Stem Cell Institute Leuven (SCIL), KU Leuven, Leuven, Belgium

[3]Department of Gastroenterology and Hepatology, University Hospitals Leuven, KU Leuven, Leuven, Belgium

[4]Department of Microbiology and Immunology, Rega Institute, KU Leuven, Leuven, Belgium.

Supplementary Figures

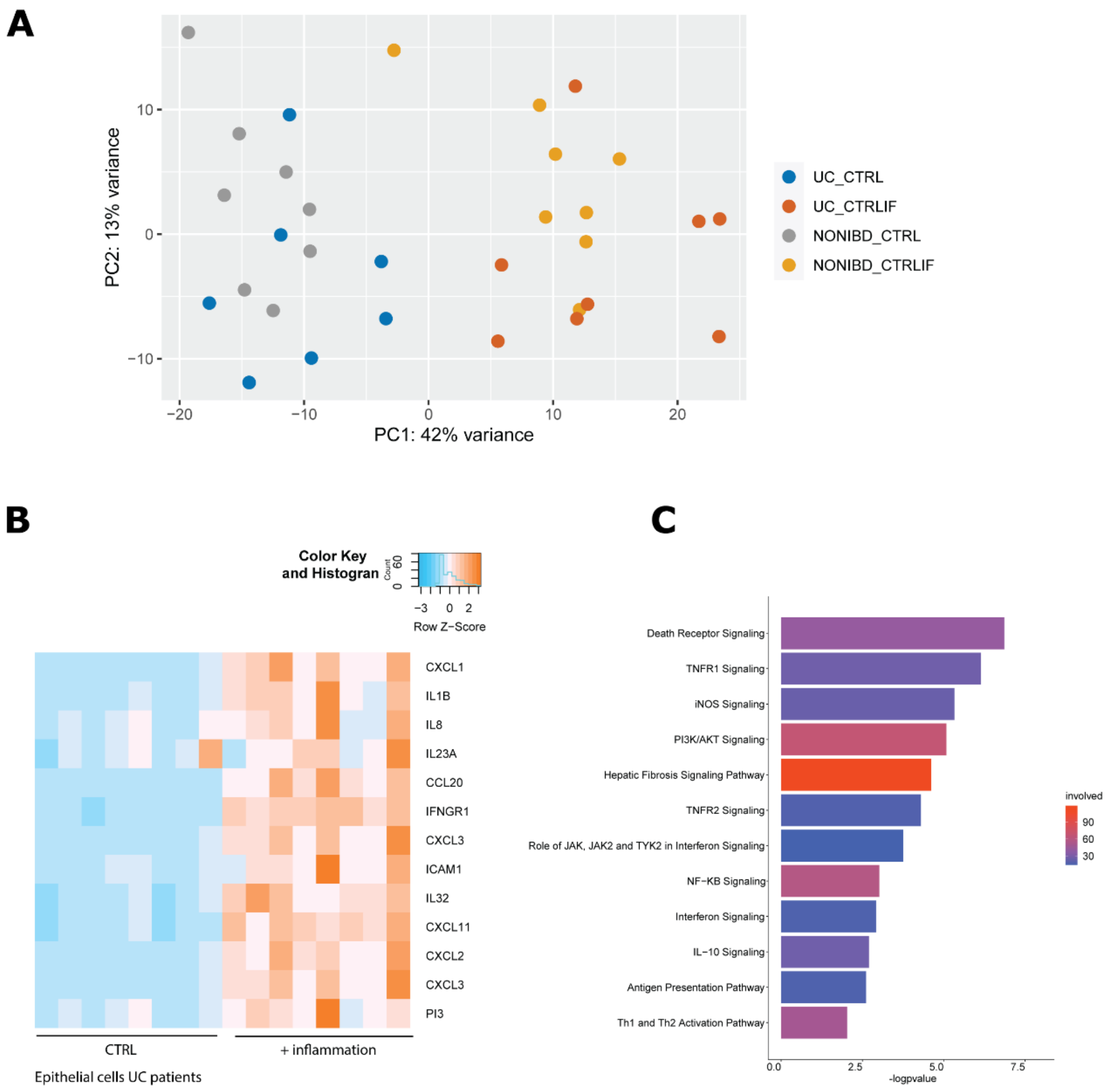

Supplementary Figure 1: (A) Stimulation with the inflammatory cytokine mix induces separate clustering of samples with and without inflammatory stimulation by principal component analysis (PCA). (B) Heatmap showing upregulation of inflammatory markers in epithelial cells of UC patients after stimulation with the inflammatory cytokine mix, compared to unstimulated UC epithelial cells. (C) Pathway analysis showing activation of inflammatory pathways in epithelial cells of UC patients following stimulation with the inflammatory cytokine mix, similar to the observed pathways in organoids after inflammatory stimulation [1]. Labels are as following: tissue_exposure; CTRL: control; IF, inflammation; NON-IBD, non-IBD control; UC: ulcerative colitis.

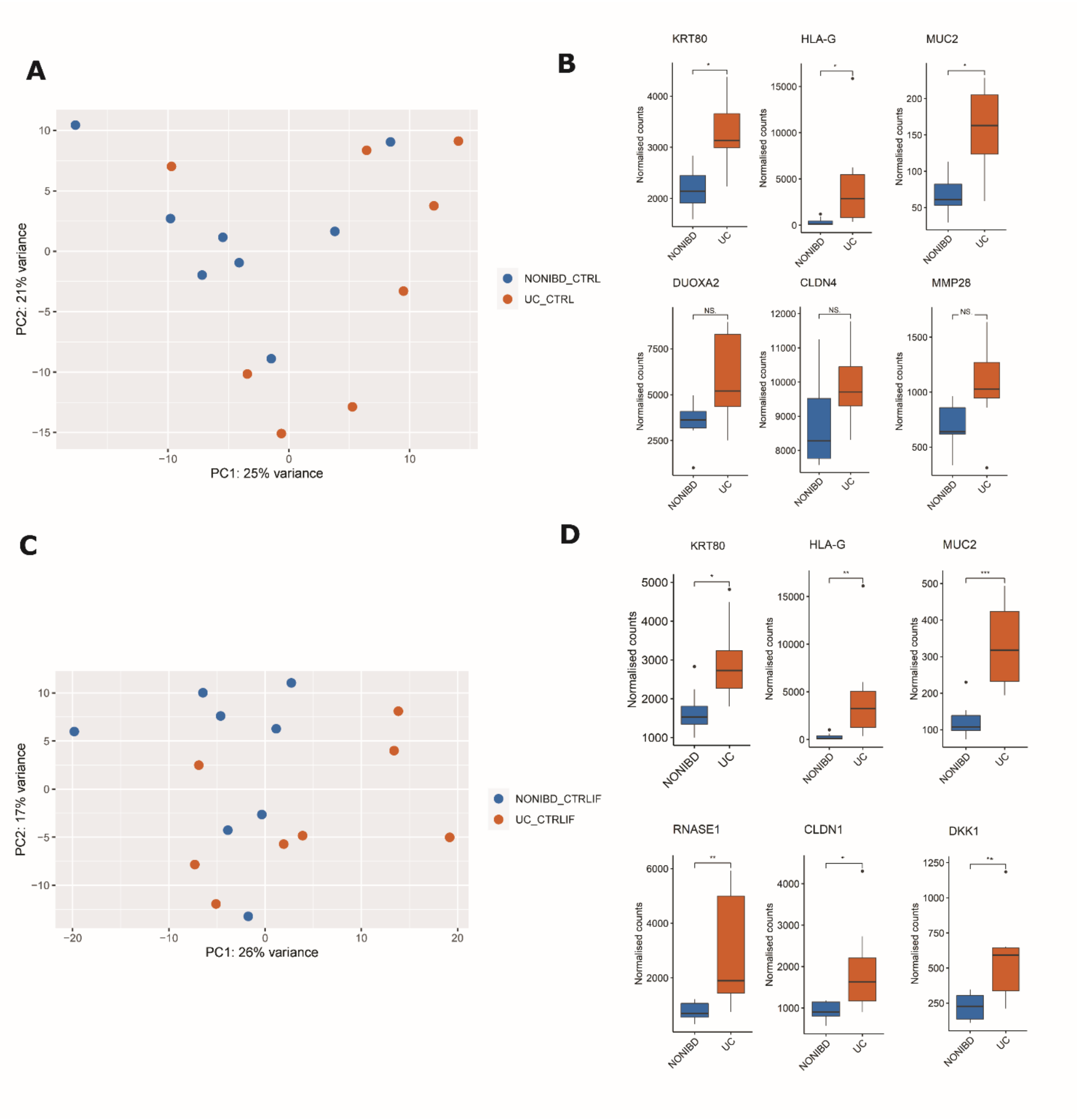

Supplementary Figure 2: (A) PCA showing NON-IBD (blue) and UC (orange) epithelial cells in control conditions. (B) Differential gene expression analysis of selected markers showing expression levels in NON-IBD and UC epithelial cells. (C) PCA showing NON-IBD (blue) and UC (orange) epithelial cells after stimulation with the inflammatory cytokine mixture. (D) Differential gene expression analysis of selected markers showing expression levels in NON-IBD and UC epithelial cells after stimulation with the inflammatory cytokine mixture. * q-value <0.05, ** q <0.01, *** q <0.001. NS, not significant. Labels are as following: tissue_exposure; CTRL: control; IF, inflammation; NON-IBD, non-IBD control; UC: ulcerative colitis.

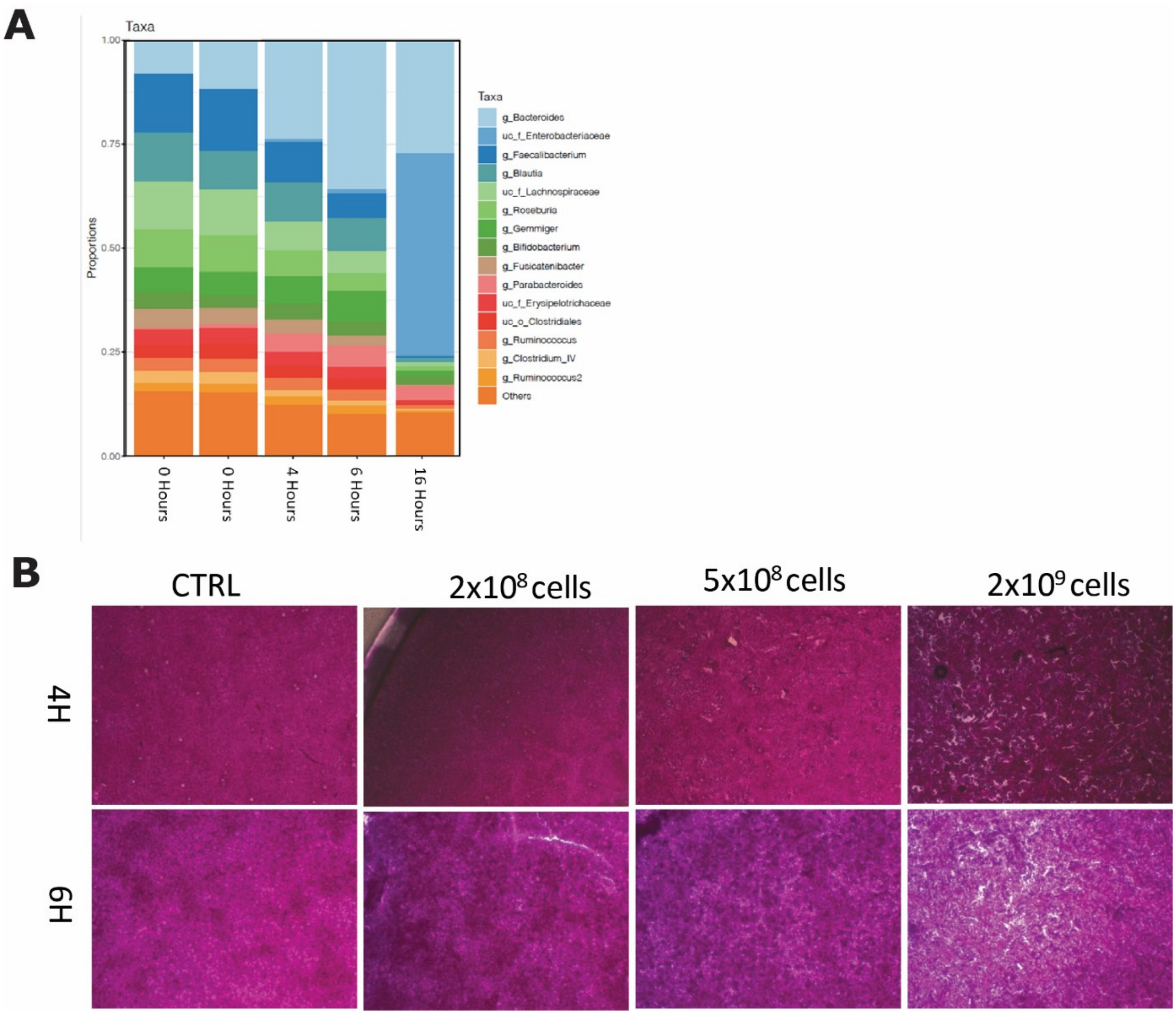

Supplementary Figure 3: Selection of conditions for co-culture experiments. (A) 16S rRNA sequencing of a microbiota sample from a healthy volunteer before co-culture and after 4, 6, and 16 hours co-culture with epithelial cells from UC patients. (B) Epithelial cells of UC patients were exposed to $2x10^8$, $3x10^8$, $5x10^8$ and $2x10^9$ microbial cells during 4 or 6 hours. Haematoxylin and eosin stain shows immediate damage to the barrier and epithelial cells for 2.10^9 cells (n=3 for all), CTRL, control; 4H, 4 hours; 6H, 6 hours.

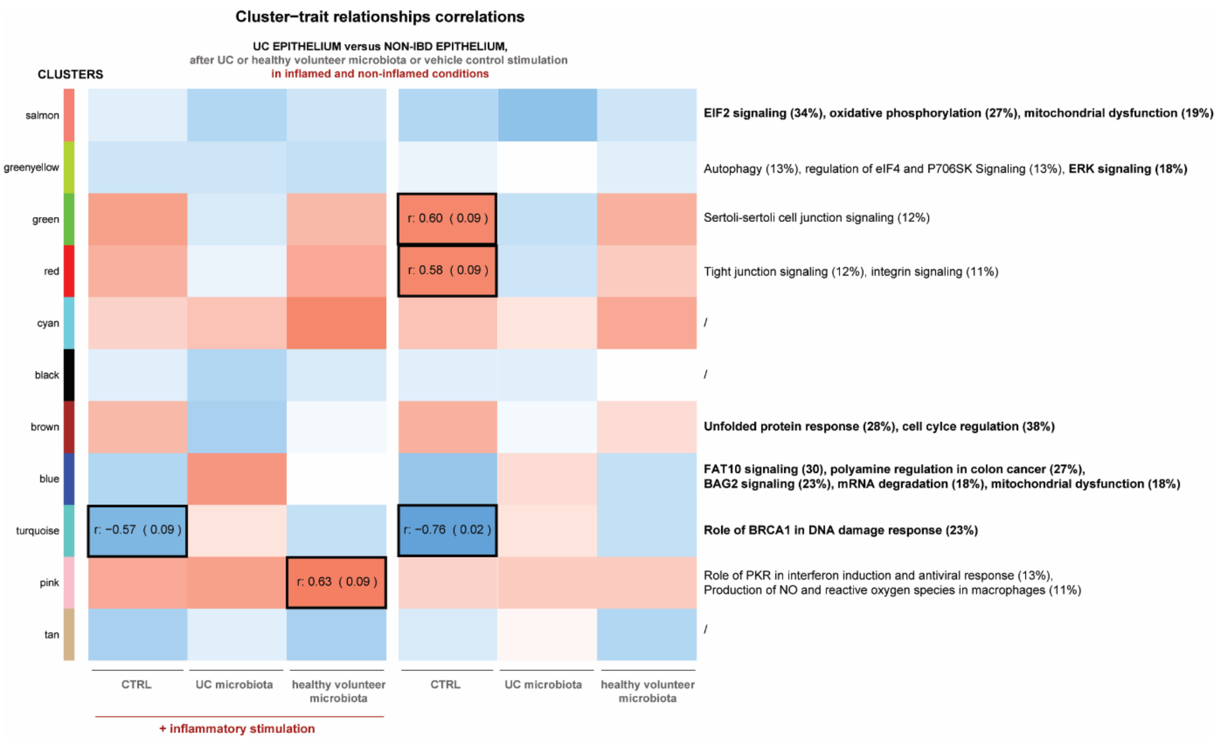

Supplementary Figure 4: Gene Co-expression Network Analysis. WGCNA was applied to compare epithelial cells from UC patients and non-IBD controls, after UC or healthy volunteer microbiota or vehicle control exposure in inflamed and non-inflamed conditions. Identified gene modules were tested for correlation with epithelial origin. Only significant correlations are highlighted (r ≥0.5 and FDR ≤0.1). Clusters indicated in red are upregulated, and in blue downregulated, in UC epithelial cells. Pathways analysis was performed for grouped modules as depicted on the figure with IPA. CTRL, control; UC, ulcerative colitis; NON-IBD, non-IBD controls; UC, ulcerative colitis.

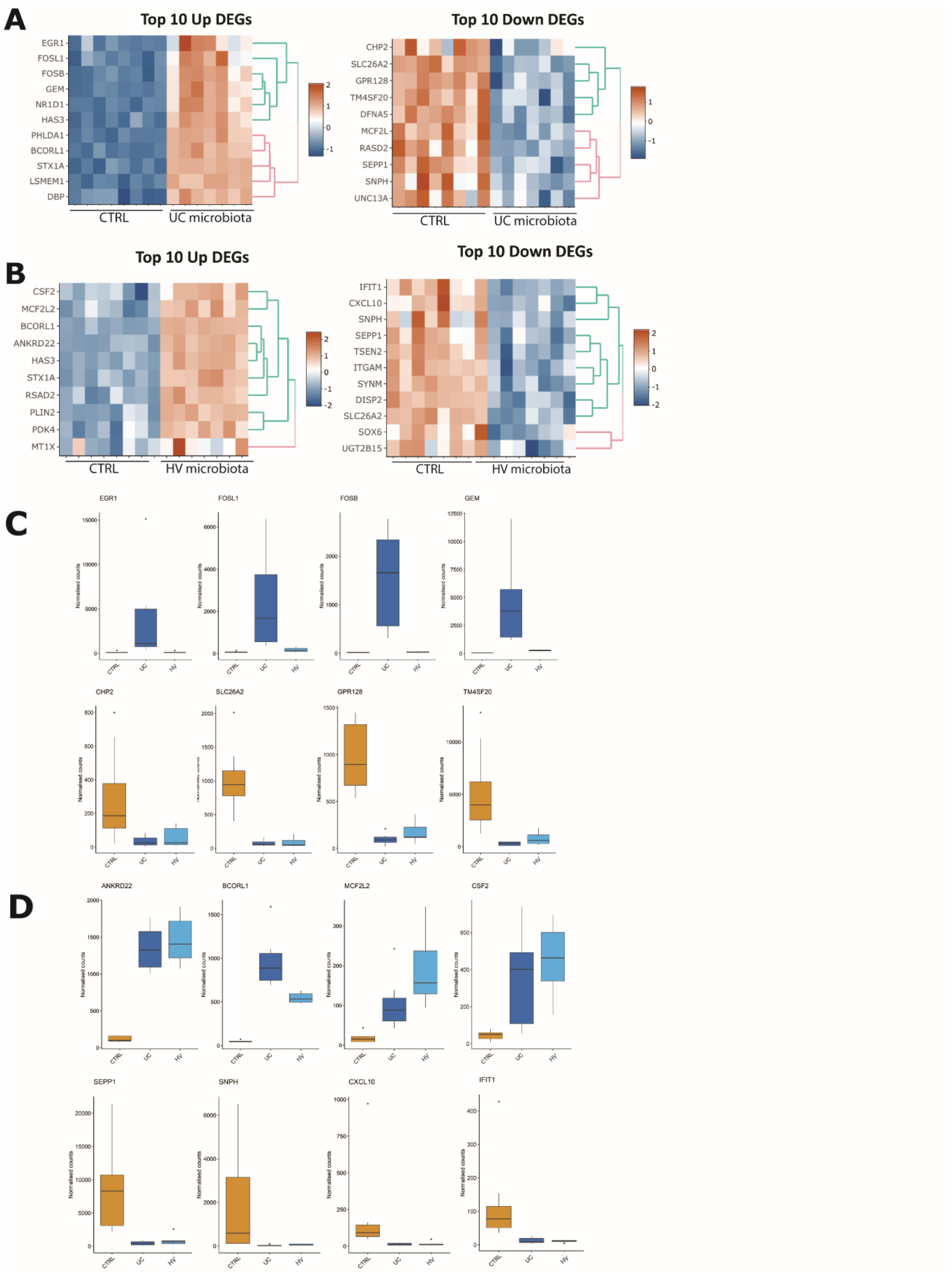

Supplementary Figure 5: (A) Top 10 up- and downregulated genes in inflamed epithelial cells of UC patients without and with exposure to healthy volunteer microbiota. (B) Top 10 up- and downregulated genes in inflamed epithelial cells of UC patients without and with exposure to healthy volunteer microbiota. UC, ulcerative colitis; NON-IBD; non inflammatory bowel disease control; HV, healthy volunteer. (C) Top up- and downregulated genes after exposure to UC in inflamed UC epithelial cells. These genes are not up- or downregulated following healthy volunteer microbiota exposure. (D) Top up- and downregulated genes after exposure to healthy volunteer microbiota in inflamed UC epithelial cells. These genes are also up- or downregulated following exposure to UC microbiota. UC, ulcerative colitis; NON-IBD; non inflammatory bowel disease control; HV, healthy volunteer.

A

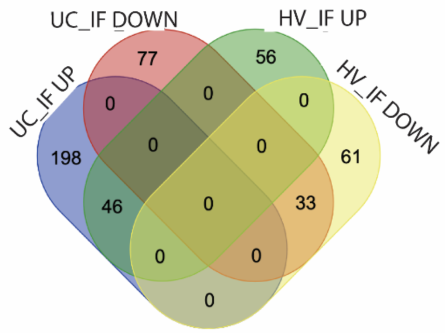

B

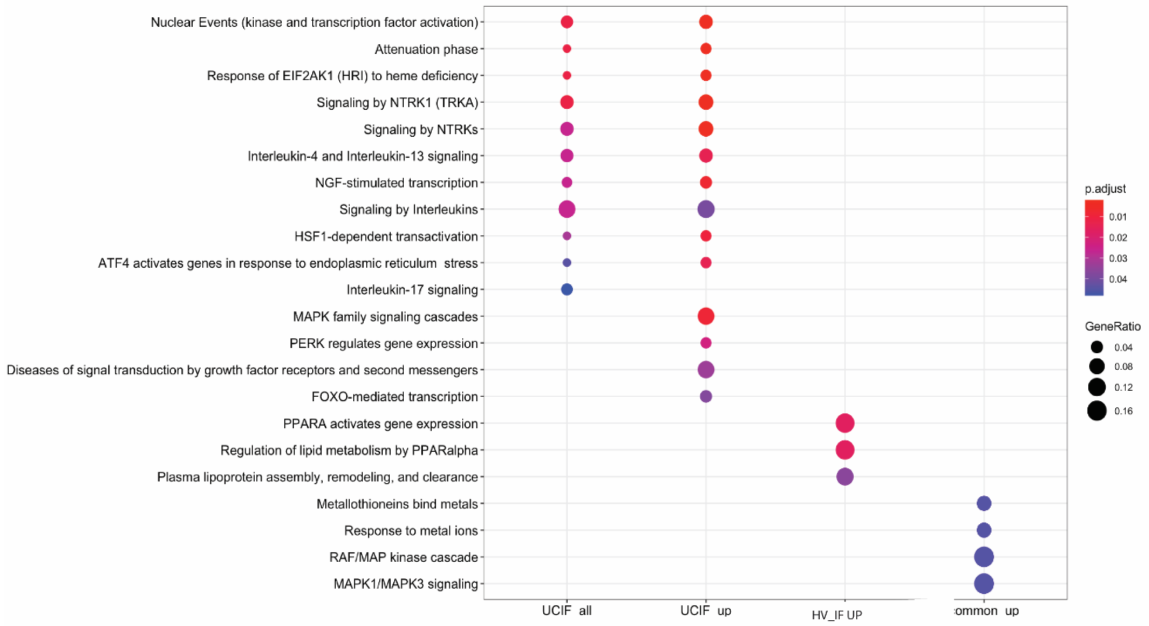

C

Source database*	Pathway Name	UC vs CTRL All DEGs	UC vs CTRL Up DEGs	UC vs CTRL Down DEGs	HD vs CTRL All DEGs	HD vs CTRL Up DEGs	HD vs CTRL Down DEGs	UC vs HD All DEGs	UC vs HD Up DEGs	UC vs HD Down DEGs
NETPATH	EGFR1			-	-	-	-	-	-	-
KEGG	MAPK signaling pathway			-	-	-	-		-	-
PID NCI	Direct p53 effectors			-	-	-	-	-	-	-
INOH	GPCR signaling			-	-	-	-	-	-	-
KEGG	Jak-STAT pathway		-	-	-	-	-	-	-	-
NETPATH	EGFR1	-	-	-	-	-	-	-	-	-
NETPATH	TGF beta Receptor	-		-	-	-	-	-	-	-

Supplementary Figure 6: (A) Visualization of the overlap and unique differentially expressed genes in inflamed UC epithelial cells following exposure to UC or healthy volunteer microbiota, compared to the control. (B) Reactome analysis showing unique and shared pathways in inflamed UC epithelial cells after exposure to UC or healthy volunteer microbiota, compared to the control. No significant downregulated pathways could be detected after exposure to healthy volunteer microbiota. (C) Analysis of additional databanks (KEGG, NETPATH, INOH) showing upregulation of pathways after exposure to UC of healthy volunteer microbiota. Significant pathways are marked in pink. CTRL, control; HV, healthy volunteer; UC, ulcerative colitis.

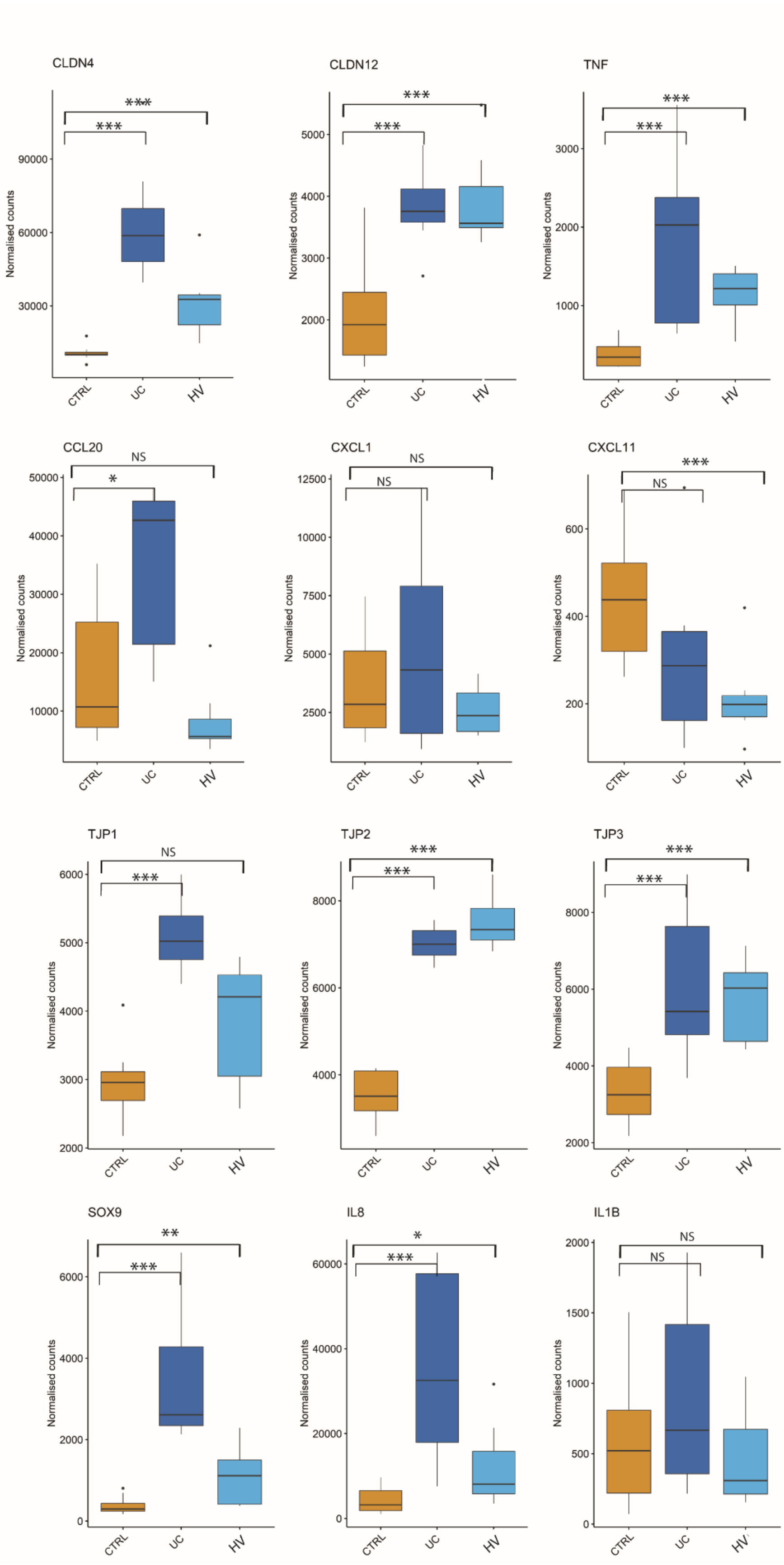

Supplementary Figure 7: Expression levels of selected inflammatory and tight junctions markers in inflamed UC epithelial cells without and with exposure to UC or healthy volunteer microbiota. FDR* <0.05, ** <0.01, ***<0.001. UC, ulcerative colitis; NON-IBD; non inflammatory bowel disease control; HV: healthy volunteer.

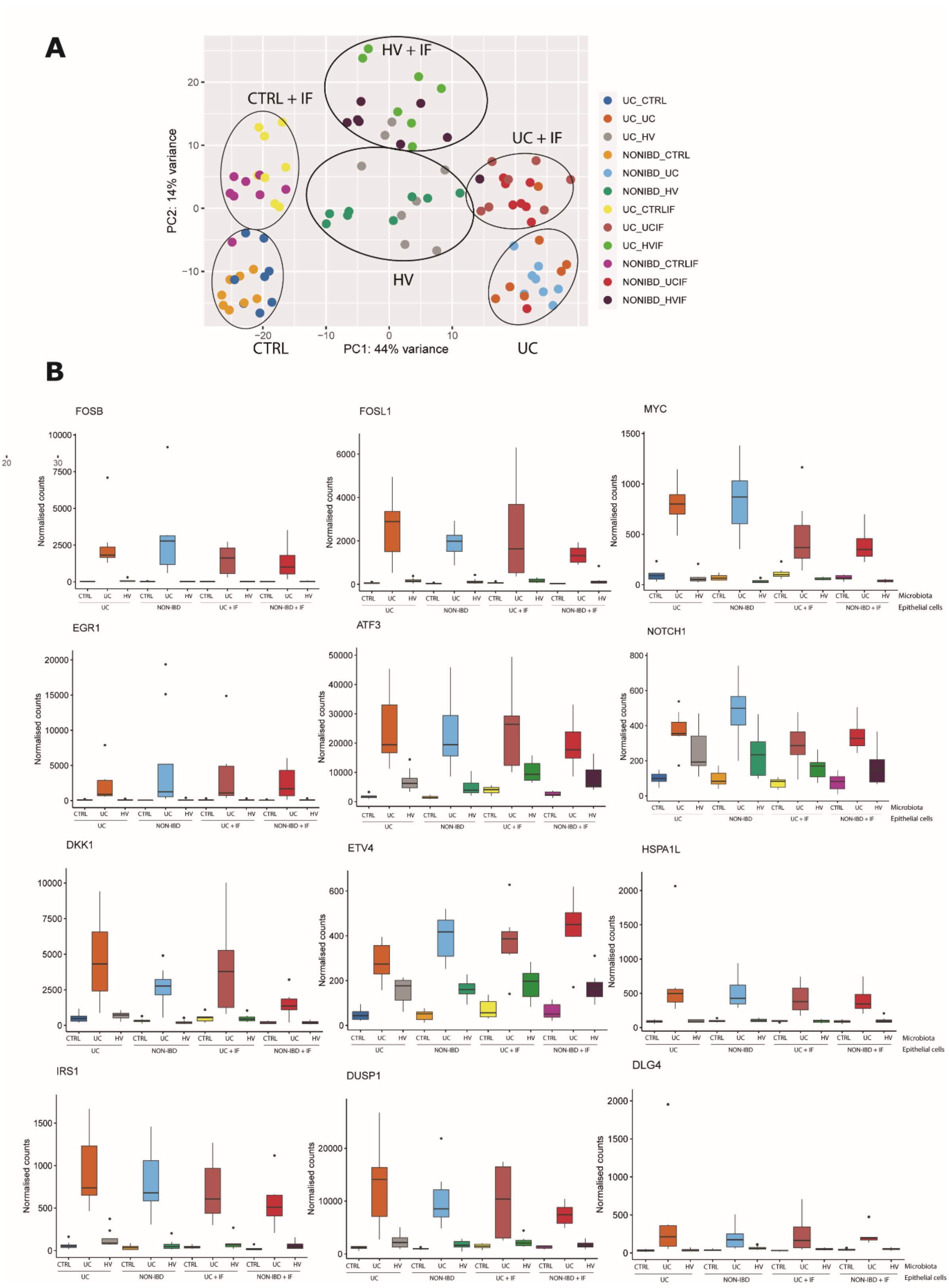

Supplementary Figure 8: (A) PCA showing clustering per microbiota stimulation, not per epithelial cell type. PC1 is driven by microbiota treatment. PC2 is driven by inflammatory stimulation. Circles are drawn to indicate the different groups. (B) Key markers observed in network analysis following UC microbiota stimulation show UC microbiota specific upregulation in all epithelial cell types. Labels are as following: tissue_exposure. UC, ulcerative colitis; NON-IBD; non inflammatory bowel disease control; HV: healthy volunteer ; IF: inflammation

Supplementary material and methods

Crypt isolation and organoid culturing

Biopsies (four per subject) were collected in basal medium (BM) and processed immediately. BM consisted of advanced DMEM:F12 supplemented with 1x GlutaMax, 10 mM HEPES and 100 U/mL penicillin, and 100 µg/mL streptomycin (Gibco, Thermo Fisher Scientific, Waltham, Massachusetts, USA).

Crypts were isolated as described before [2]. In short, biopsies were washed thoroughly in chelating solution (5.6 mM Na_2HPO_4, 8.0 mM KH_2PO_4, 96.2 mM NaCl, 1.6 mM KCl, 43.4 mM sucrose, 54.9 mM D-sorbitol, 0.5 mM DL-dithiothreitol). Next, biopsies were incubated for 45 minutes in chelation solution supplemented with 2mM EDTA (Sigma-Aldrich, Missouri, USA) at 4°C on a rocking platform. Thereafter, biopsies were disrupted by rigorously pipetting with chelation solution to loosen and collect the crypts. The crypts were embedded in Matrigel (phenol red free, growth factor reduced, Corning, New York, USA) diluted by 50 % BM. Organoids were cultured in human expansion medium (HM) (BM supplemented with growth factors, **Supplementary table 3**) and split mechanically every seven days as previously described [3].

Transwell® cultures

Transwell® cultures were cultured as described before [3]. In brief, organoid cultures were dissociated and transferred to Transwell® inserts 2-3 days after splitting. Transwell® inserts (3470, Corning Costar) were coated with 0.1 mg/mL collagen type I [rat tail, Corning] for 24h at 37°C. Organoids (three wells of a 24 well plate for one Transwell®) were collected with ice cold BM. Organoids were subsequently mechanically splitted, treated with 0.5 mM EDTA (Sigma-Aldrich) and incubated with 0.25 % Trypsin/EDTA (Gibco) including five minutes at 350 G centrifugation between each step. Fractions were further mechanically dissociated into small clumps of cells and seeded in the apical compartment of the Transwell®. Cells were cultured for the first 24 hours in 50 % HM [50 % BM, 50 % HM] supplemented with 10 µM Y-27632 (ROCK inhibitor; Tocris Bioscience and R&D Systems; Minneapolis, USA). After 24 hours, monolayers were cultured in 50 % HM with medium changes every other day. Before initiating further analysis, monolayers were grown until confluency (approximately seven days) as

monitored by daily Transepithelial electrical resistance (TEER) measurements. TEER was also measured (in duplicate) at the start and end of each experiment.

Haematoxylin and Eosin Stain

Transwells® were washed once with PBS, followed by five minutes fixation with chilled methanol. The cultures were washed with distillated H2O, stained for one minute with 1 % Eosin, washed three times with distilled H2O, stained for four minutes with Haematoxylin, washed once in distilled H2O and washed twice with tap water. For imaging, the Transwells® were transferred to a new plate with fresh tap water and imaged with a Zeiss Axiovert 40 CFL microscope (Carl Zeiss Inc, Oberkochen, Germany) and an AxioCam Mrc5 camera (AxioVision Rel 4.8 software).

FITC-dextran (4 kD) permeability measurements

Transwells® were incubated for one hour at 37°C with 2 mg/mL FITC-dextran beads (4 kD, Merck, New Jersey, USA) at the apical side. After one hour, the concentration in the basolateral medium was measured in triplicate by a plate reader (FLUOstar Omega, BMG Labtech) at an excitation wavelength of 485 nm and an emission wavelength of 530 nm. The concentration of migrated beads in the control condition was considered as 100 %.

RNA extraction

Transwells® were washed once with PBS. Next, cultures were incubated for five minutes with 0.25% Trypsin-EDTA. The monolayers were mechanically dissociated and collected with BM with 10% FBS to stop the trypsinisation. The pellet was washed once with PBS before addition of lysis buffer and 12 mM β-mercapto-ethanol. RNA extraction was performed using the Promega ReliaPrep™ miRNA Cell and Tissue Miniprep System. RNA was quantified by Nanodrop (Thermo Scientific, Waltham, MA, USA) and the integrity was evaluated using Agilent Bio-analyzer 2100 (Agilent, Waldbronn, Germany). A minimal RIN of eight was required for downstream analysis.

RT-qPCR

cDNA was synthesized from normalized RNA quantities using SuperScript™ III First-Strand Synthesis SuperMix (Invitrogen, Carlsbad, USA). Real time quantitative PCR (RTqPCR) was performed using Platinum SYBR green qPCR Supermix-UDG (Invitrogen) with primers from Integrated DNA Technology (Idt, San Jose, CA) and run on a Viia 7™ system (Applied Biosystems, Foster City, CA). Samples were run in duplicate and analysed using the ΔΔCt

method with normalization to the geometric mean of two reference genes (Ribosomal protein S14 *(RPS14)* and Hypoxanthine Phosphoribosyltransferase 1 *(HPRT1)*). The primer sequences for CXCL1, DUOXA2, IL1β, IL8, OCLN, TLR4, TNFα, ZO1 can be found in **Supplementary table 4**.

WGCNA

To identify phenotypically relevant network clusters (modules) based on highly correlated genes, weighted gene co-expression network (WGCNA) was performed on the count filtered normalized dataset, as described earlier [4, 5]. Pearson correlation co-efficient was used as a measure to quantify the co-expression relationship for a given gene-pair. The module eigengene was defined as the first principal component summarizing the expression patterns of all genes into a single expression profile within a given module. Genes showing the highest correlation with the module eigengene were referred to as hub genes. Phenotypically relevant clusters were identified using cluster-trait correlations ($|r| > 0.5$, FDR ≤ 0.1).

Functional enrichment analysis

In addition to Reactome and Gene Ontology terms, KEGG (Kyoto Encyclopedia of Genes and Genomes)[6], PID NCI (Pathway Interaction Database) [7], INOH (Integrating Network Objects with Hierarchies database) [8] and NETPATH [9] were used as reference databases.

Regulatory network construction

Transcription factors (TFs) capable of physically binding (as determined using ChIP-Seq evidence gathered from ENCODE, ReMap and literature) to the cis-elements of the identified DEGs or co-expressed network genes (from GTEx and ARCHS4) were identified from the ChEA3 resource [10]. To focus on the core genes of the network clusters, a trimmed version of the phenotypically relevant modules was inferred by using a minimum gene-eigengene correlation filter ($|r| > 0.7$).

A transcriptional regulatory interaction was assigned between a TF and a target gene (TG) for a comparison if the following three criteria were met: (a) both the TF and its targets are DEGs (b) TF is capable of binding to the cis-elements of the TG as inferred by ChIP-Seq evidence and (c) TF and TG are co-expressed as inferred from the WGCNA analysis. As an additional annotation (not a requirement), TF-TG relationships satisfying the above three criteria were also annotated using the co-expression data retrieved from GTEx and ARCHS4. Regulon (set of target genes regulated by a TF) wise functional enrichment analysis was performed using

the same criteria as recorded in the previous section. Network visualizations were performed using Cytoscape version 3.8.2 [11].

16S rRNA sequencing

16S rRNA sequencing of microbial samples was performed before and after co-culture with epithelial cells. The MagAttract® PowerMicrobiome® DNA/RNA KF Kit (Qiagen) was used to extract DNA from the microbial samples. The V4 variable region of the 16S rDNA was amplified using dual index primer pairs 515F and 806R (GTGYCAGCMGCCGCGGTAA and GGACTACNVGGGTWTCTAAT, respectively). Sequencing was performed using the Illumina MiSeq platform with sequencing kit MiSeq v2, producing 250bp paired-end reads. Size selection was performed using Agencourt AMPure to remove fragments below 200 bases. Sequencing was carried out on the Illumina HiSeq platform at the VIB Nucleomics core laboratory (Leuven, Belgium) with 500 cycles (sequencing kit HiSeq-Rapid SBS kit, version 2), producing 2x 250bp paired-end sequencing reads. After de-multiplexing with sdm as part of the LotuS pipeline [12] without allowing for mismatches, fastq sequences were further analysed per sample using DADA2 pipeline (v. 1.6) [13] .

Supplementary tables

Supplementary table 1: baseline characteristics of the study population.

**Baseline characteristics**	**Biopsies**	**Biopsies**	**Faecal samples**
	**UC**<br>**(n=8)**	**Non-IBD controls**<br>**(n=8)**	**UC**<br>**(n=2)**
Male/Female (%)	5/3 (62.5/37.5)	3/5 (37.5/62.5)	2/0 (100/0)
Median (IQR) age at endoscopy (years)	46.87 (39.46-54.93)	46.40 (37.80-57.37)	32.23 (29.81-34.66)
Median (IQR) disease duration (years)	15.37 (11.25-15.50)	N/A	13.5 (13.25-13.75)
**Endoscopic Mayo sub-score**			
Mayo 2 (%)	4 (50)	N/A	1 (50)
Mayo 3 (%)	4 (50)		1 (50)
**Organoids information**			
Passage	5.12 (4.25-6)	5.87 (5.25-6)	N/A
Days on transwell until microbiota	6.75 (6-7.75)	6.25 (5-7)	
**Therapy at moment of endoscopy**			
5-Aminosalicylates (%)	3 (37.5)	N/A	1 (50)
Corticosteroids (%)	1 (12.5)		0 (0)
Thiopurines (%)	1 (12.5)		0 (0)
Biological therapy (%)	0 (0)		0 (0)
Other (%)	0 (0)		0 (0)
**Indication for endoscopy**			
Ulcerative colitis (%)	8 (100)	0 (0)	2 (100)
Polyp screening (%)	0 (0)	6 (75)	0 (0)
Aspecific gastrointestinal complaints	0 (0)	2 (25)	0 (0)

IBD: inflammatory bowel disease; IQR: interquartile range; N/A: not applicable; UC: ulcerative colitis

Supplementary table 2: excluded samples from RNA sequencing analysis

Patient	Condition	Exclusion reason
UC patient 1	UC microbiota	Low quality reads
UC patient 7	Healthy volunteer microbiota + inflammatory stimulation	Low quality reads
UC patient 8	UC microbiota + inflammatory stimulation	Low quality reads

Supplementary table 3: Human medium (HM)

Component	Concentration	Manufacturer	Catalogue number
Wnt3A	50 % v/v	In house cell line	
R-spondin	20 % v/v	In house cell line	
Noggin	10 % v/v	In house cell line	
EGF	50 ng/mL	Life Technologies	PMG8041
A83-01	500 nM	Tocris	2939/10
SB202190	10 µM	Sigma-Aldrich	S7067
Nicotinamide	10 mM	Sigma-Aldrich	N0636
n-Acetylcysteine	1.25 mM	Sigma-Aldrich	A9165
B27	1x	Life Technologies	17504044

v/v: percentage volume of total end volume

Supplementary table 4: primer sequences

Gene		Forward primer (5'-3')	Reverse primer (5'-3')
**RPS14**	Ribosomal protein S14	TCACCGCCCTACACATCAAAC	GCCCGATCTTCATACCCGA
**HPRT1**	Hypoxanthine Phosphoribosyltransferase 1	GAAAAGGACCCCACGAAGTGT	AGTCAAGGGCATATCCTACAACA
**CXCL1**	chemokine (C-X-C motif) ligand 1	CATCCCCCATAGTTAAGAAAATCATC	TCTCTCTTTCCTCTTCTGTTCCTA
**DUOXA2**	Dual oxidase maturation factor 2	CCACTGCTCATCGTTATTCTAGT	GTGCACAGCCACAATTTCTG
**IL1B**	Interleukin-1-beta	TTCGACACATGGGATAACGAGG	TTTTTGCTGTGAGTCCCGGAG
**IL8**	Interleukin-8	ACTGAGAGTGATTGAGAGTGGAC	AACCCTCTGCACCCAGTTTTC
**OCLN**	Occludin	GCAGGAAGGTCAAAGAGAACAGA	ATATTCCCTGATCCAGTCCTCCTC
**TLR4**	Toll like receptor 4	AGTTGATCTACCAAGCCTTGAGT	GCTGGTTGTCCCAAAATCACTTT
**TNFA**	Tumour necrosis factor alpha	TAGCCCATGTTGTAGCAAACCC	TATCTCTCAGCTCCACGCCA
**ZO1**	Zona occludens 1	CAGTGCCTAAAGCTATTCCTGTGA	CTATGGAACTCAGCACGCCC

Supplementary references

1. Arnauts K, Verstockt B, Ramalho AS, et al. Ex Vivo Mimicking of Inflammation in Organoids Derived From Patients With Ulcerative Colitis. Gastroenterology 2020;159:1564-1567.
2. Vanhove W, Nys K, Arijs I, et al. Biopsy-derived Intestinal Epithelial Cell Cultures for Pathway-based Stratification of Patients With Inflammatory Bowel Disease. J Crohns Colitis 2018;12:178-187.
3. Vancamelbeke M, Laeremans T, Vanhove W, et al. Butyrate does not protect against inflammation-induced loss of epithelial barrier function and cytokine production in primary cell monolayers from patients with ulcerative colitis. J Crohns Colitis 2019.
4. Langfelder P, Horvath S. WGCNA: an R package for weighted correlation network analysis. BMC Bioinformatics 2008;9:559.
5. Verstockt B, Verstockt S, Abdu Rahiman S, et al. Intestinal Receptor of SARS-CoV-2 in Inflamed IBD Tissue Seems Downregulated by HNF4A in Ileum and Upregulated by Interferon Regulating Factors in Colon. J Crohns Colitis 2021;15:485-498.
6. Kanehisa M, Sato Y, Kawashima M, et al. KEGG as a reference resource for gene and protein annotation. Nucleic Acids Res 2016;44:D457-62.
7. Schaefer CF, Anthony K, Krupa S, et al. PID: the Pathway Interaction Database. Nucleic Acids Res 2009;37:D674-9.
8. Yamamoto S, Sakai N, Nakamura H, et al. INOH: ontology-based highly structured database of signal transduction pathways. Database (Oxford) 2011;2011:bar052.
9. Kandasamy K, Mohan SS, Raju R, et al. NetPath: a public resource of curated signal transduction pathways. Genome Biol 2010;11:R3.
10. Keenan AB, Torre D, Lachmann A, et al. ChEA3: transcription factor enrichment analysis by orthogonal omics integration. Nucleic Acids Res 2019;47:W212-W224.
11. Shannon P, Markiel A, Ozier O, et al. Cytoscape: a software environment for integrated models of biomolecular interaction networks. Genome Res 2003;13:2498-504.
12. Hildebrand F, Tadeo R, Voigt AY, et al. LotuS: an efficient and user-friendly OTU processing pipeline. Microbiome 2014;2:30.
13. Callahan BJ, McMurdie PJ, Rosen MJ, et al. DADA2: High-resolution sample inference from Illumina amplicon data. Nat Methods 2016;13:581-3.
